# Production and Characterization of Kefir Beverages by Fermentation of Whole Milk with Milk or Water Kefir Grains

**DOI:** 10.3390/foods15101616

**Published:** 2026-05-07

**Authors:** Eduardo Balvis Outeiriño, Marta Abajo Justel, Cristina Pérez Novo, Alberto Acuña Couñago, Nelson Pérez Guerra

**Affiliations:** 1 Department of System Engineering and Automatic, Faculty of Sciences, University of Vigo, Ourense Campus, As Lagoas s/n, 32004 Ourense, Spain; ebalvis@uvigo.gal; 2 Industrial Biotechnology and Environmental Engineering Group “BiotecnIA”, Department of Analytical and Food Chemistry, University of Vigo, Ourense Campus, As Lagoas s/n, 32004 Ourense, Spain; venexoc@gmail.com; 3 Centro de Apoio Científico e Tecnolóxico a la Investigación (CACTI), University of Vigo, Auga Campus, Rua Canella da Costa da Vela 12, 32004 Ourense, Spain; cperezn@uvigo.es; 4 Centro de Apoio Científico e Tecnolóxico a la Investigación (CACTI), University of Vigo, As Lagoas Campus, Marcosende s/n, 36310 Vigo, Spain; berto@uvigo.es

**Keywords:** kefir beverages, milk kefir grains, water kefir grains, kinetics analysis, microbiological composition, chemical and volatile profile

## Abstract

This study evaluated the fermentation kinetics and properties of kefir beverages produced from whole milk using milk kefir grains (MKGs) or water kefir grains (WKGs) over 48 h. MKGs showed higher initial microbial loads and promoted rapid acidification, with pH decreasing from 6.70 to 4.99 and significant production of lactic acid (10.58 g/L) and ethanol (5.17 g/L), compared with WKGs (final pH 6.20, <0.5 g/L lactic acid, and <0.3 g/L ethanol). However, the final concentration of acetic acid in WKG fermentation (1.93 g/L) was comparable to that in MKG fermentation (2.02 g/L). Microbial populations increased in both systems, exceeding 10^6^ CFU/mL—one of the requirements for conferring probiotic relevance to a beverage—with MKGs reaching higher counts. Lactose and protein consumption were greater in MKGs, suggesting more intense metabolic activity. Fermentation enhanced nutritional value by increasing vitamins B2, B3, B5, and pyridoxine, while vitamin D3 decreased. Mineral composition remained largely unchanged. Volatile analysis identified 31 compounds: MKGs favored fatty acids and lactones associated with creamy notes, whereas WKGs promoted ester formation and fruity aromas. Overall, both grain types produced microbiologically safe beverages with distinct biochemical and sensory profiles, demonstrating the feasibility of using WKGs for milk fermentation.

## 1. Introduction

The production and consumption of kefir and kefir-like beverages have increased considerably in recent years, largely due to the rising interest in functional foods and fermented products with potential health-promoting properties. Kefir is widely recognized as a functional beverage, and numerous studies have reported a variety of beneficial biological activities associated with its consumption. These include anti-inflammatory, antioxidant, antitumoral, and antidiabetic effects, among others [1,2,3,4,5]. Additionally, through the production of multiple antimicrobial compounds—including organic acids, alcohols, hydrogen peroxide, diacetyl, and bacteriocins—the microbiota present in milk kefir grains (MKGs) and water kefir grains (WKGs) exhibit antibacterial activities against important foodborne and pathogenic bacteria and yeasts [1,2].

Such health-related properties have contributed to the expanding global demand for kefir and kefir-derived products, as well as to the increasing scientific interest in understanding their microbiological composition, biochemical transformations, and functional characteristics [6].

Traditionally, kefir is produced using various types of milks (e.g., cow, goat, sheep, camel, or buffalo) as the substrate and MKGs as the fermentation agent. MKGs harbor a diverse microbiota composed of different species of lactic acid bacteria (LAB), acetic acid bacteria (AAB), and yeasts that coexist in a stable symbiotic relationship. During fermentation, this microbial community metabolizes milk nutrients, producing biochemical changes that significantly influence the physicochemical characteristics and sensory properties of the fermented beverage, while also contributing to its functional properties and improved digestibility [3,4,6,7,8,9,10,11]. As a result, kefir is generally characterized as a mildly acidic beverage with reduced lactose content compared with the unfermented substrate and low alcohol levels, typically below 1–2%. It also exhibits distinctive sensory attributes, including a slightly effervescent texture, creamy consistency, and a complex aroma profile. These characteristics distinguish kefir from fermented foods produced using less complex inocula (e.g., yogurt) and contribute to its growing popularity among consumers seeking functional foods with probiotic potential [3,4,10,11,12].

Several studies have demonstrated the feasibility of producing innovative kefir-like functional beverages by fermenting non-dairy substrates [13,14,15,16,17,18,19] using MKGs. This approach addresses current trends, such as the growing demand for plant-based foods, as well as the need to provide alternatives for individuals with lactose intolerance or milk allergies.

Another approach for producing non-dairy fermented beverages involves the use of WKGs to ferment sugar-rich aqueous solutions, often supplemented with fruits or plant extracts, resulting in lightly carbonated beverages with mild acidity and fruity flavors, highlighting their potential as novel functional drinks [13,20,21].

Although both MKGs and WKGs contain LAB, AAB, and yeasts, the composition and relative abundance of microbial species differs significantly between the two types of grains [4,22]. Consequently, fermenting the same substrate with MKGs or WKGs may yield beverages with distinct microbiological, chemical, and sensory characteristics, as observed by Zongo et al. [13].

Despite the increasing interest in kefir fermentation systems and the use of a wide range of alternative substrates, a key premise for the present study is that milk fermentation using WKG has not yet been systematically investigated. Exploring this possibility could provide new insights into the adaptability of WKG microbiota to a lactose-containing substrate and help clarify how different microbial consortia influence fermentation processes and product characteristics. Thus, the production of a fermented milk beverage using WKG could expand the range of fermented dairy products, support product diversification, and offer potential opportunities for commercialization in the dairy industry.

Therefore, this study aimed to elucidate, for the first time, the kinetics of milk fermentation using WKG for the production of a novel kefir-like beverage. The fermentation process was monitored by evaluating the time course of several parameters, including changes in culture pH, the growth dynamics of LAB, AAB, and yeasts, the consumption of lactose and proteins, and the production of organic acids, alcohols, and antibacterial activity. The concentrations of minerals, vitamins, and volatile compounds in beverages obtained after 24 and 48 h of incubation were also quantified and compared. For comparison purposes, the same experimental approach was applied using traditional MKGs, allowing a direct assessment of differences between the two fermentation systems.

## 2. Materials and Methods

### 2.1. Inoculum Preparation

MKGs and WKGs were purchased from Kefiralia (Burumart Commerce S.L., Arrasate, Spain) and stored at 4 °C until activation.

MKGs were activated in fresh ultra-high temperature (UHT) whole milk (Central Lechera Asturiana, Asturias, Spain) [23]. The mean composition of the milk was as follows: lactose, 46.84 ± 2.18 g/L; proteins, 31.99 ± 0.06 g/L; total nitrogen, 5.40 ± 0.01 g/L; and pH, 6.70 ± 0.03.

All materials (e.g., plastic spoons, knives, and strainers) and components (sugar, salt, bottled water, milk, lemon, and dates) used in the activation and fermentation media were placed in a biosafety cabinet and exposed to ultraviolet light for 30 min to reduce surface microbial load and ensure aseptic handling conditions.

For activation, WKGs, provided without a covering liquid, were placed into a previously sterilized 3.1 L glass jar containing 1 L of a sugar solution (40 g sucrose/L of mineral water, Cabreiroá, Ourense, Spain), supplemented with a pinch of NaCl, two dates, and one slice of lemon, as recommended by the supplier. The incubation conditions (room temperature, without agitation for 24 h, avoiding direct sunlight) were similar to those used for MKG activation. This activation step was repeated six times.

UHT whole milk, sucrose (Hacendado, Mercadona, Spain), mineral water (Cabreiroá, Ourense, Spain), salt (Hacendado, Mercadona, Spain), branch dates (Hacendado, Mercadona, Spain), and lemons (Frutas Poveda S.A., Murcia, Spain) were purchased from a local supermarket (Mercadona S.A., Ourense, Spain).

### 2.2. Fermentation Conditions and Sampling

Kefir beverages were produced by fermenting UHT whole milk with either MKGs or WKGs. Fermentation with each type of grain was carried out in duplicate using 18 clean, sterilized 140 mL glass jars, each containing 50 mL of substrate and inoculated with 1.5 g of kefir grains. This inoculation rate (0.03 g of kefir grains per mL of substrate) was previously identified as optimal for achieving the highest viable cell counts of LAB, AAB, and yeasts in kefir-like drinks produced from kiwifruit juice fermented with MKG over three consecutive 24 h subcultures [18]. Although this inoculation rate may not be optimal for milk fermentations with both grain types, it allows comparison with results obtained from kiwifruit juice fermentations.

The fermentation conditions were the same as those used for the activation of both types of grains. At regular intervals (4, 8, 12, 16, 20, 24, 28, 32, and 48 h), duplicate jars from each fermentation (MKG or WKG) were collected to measure culture pH, kefir grain weight, and the concentrations of lactose, protein, organic acids, and alcohols. A previous study [23] showed that fermenting milk with MKGs for 48 h reduced the total concentration of volatile compounds and led to whey separation, curd formation, and the formation of large gas bubbles, all of which negatively affected the beverage’s visual appearance.

Although there is no direct evidence in the literature of these effects occurring during milk fermentation with WKGs, and given that the fermentation kinetics of milk with MKGs were used as a control in the present study, a maximum fermentation time of 48 h was also applied to WKG fermentations. This approach ensured consistency and enabled comparison between both systems.

From each jar, two 5 mL aliquots (A and B) of fermented milk were withdrawn and used for microbiological analysis and determination of proteins (aliquot A), and total antibacterial activity (aliquot B). The remaining sample was centrifuged at 12,000× *g* for 15 min at 4 °C. After measuring the pH of the supernatant, it was divided into two aliquots (C and D; 30 and 8 mL, respectively) and stored at −40 °C for subsequent determination of sugars, organic acids, alcohols, vitamins, mineral content (aliquot C), and volatile compounds (aliquot D).

Viable cell counts of LAB, AAB, and yeasts, as well as total antibacterial activity (TAA), were determined in unfermented milk and in samples collected at 8, 16, 24, 32, and 48 h. Concentrations of minerals, vitamins, and volatile organic compounds (VOCs) were quantified in unfermented and fermented beverages obtained after 24 and 48 h of incubation (MKG-24 h, MKG-48 h, WKG-24 h, and WKG-48 h).

### 2.3. Viable Cell Counts in MKGs, WKGs, and the Fermentation Medium

The viable cell counts of LAB, AAB, and yeasts in MKGs and WKGs were determined in this study as described by Bazán et al. [17] and expressed as log colony-forming units (CFUs) per gram of wet kefir grains.

For the microbiological characterization of unfermented and fermented substrates, duplicate samples of whole milk (0 h) and aliquot A were serially diluted and plated in triplicate to enumerate mesophilic LAB, AAB, yeasts, Enterobacteriaceae, and *Pseudomonas* spp. The first three groups of microorganisms were counted using MRS agar, Carr agar (both supplemented with amphotericin B, 0.1 g/L), and yeast extract–glucose (YEG) agar supplemented with chloramphenicol (0.1 g/L), respectively [17,24]. Enterobacteriaceae and *Pseudomonas* spp. were enumerated using violet red bile glucose agar (VRBGA, double-layered) and *Pseudomonas* agar base (PAB) supplemented with cetrimide–fucidin (10 g/L), respectively [17,24].

Plates for LAB, AAB, yeasts, Enterobacteriaceae, and *Pseudomonas* were incubated as described in previous studies [17,24]. For clarity, results were expressed as log CFU/mL of substrate.

MRS, PAB, and YEG media were purchased from Panreac Química S.A. (Barcelona, Spain), while VRBGA and Carr medium were supplied by Oxoid (Milan, Italy) and Condalab Laboratories S.A. (Madrid, Spain), respectively. Chloramphenicol and amphotericin B were purchased from Cientisol (A Coruña, Spain).

### 2.4. Chemical Composition of Substrate and Fermented Beverages

The contents of lactose, organic acids (lactic and acetic acids), and alcohols (ethanol and glycerol) in both whole milk and fermented beverages were analyzed in triplicate for each sample using high-performance liquid chromatography (HPLC), as previously described [17]. First, unfermented and fermented samples (aliquot C) were filtered through hydrophilic polytetrafluoroethylene syringe filters (0.2 μm pore size, Fisher Scientific S.L., Madrid, Spain). During HPLC analysis, a refractive index detector and a diode array detector were connected in series to rule out potential metabolite co-elution. Standard solutions of lactose, lactic acid, acetic acid, ethanol, and glycerol, at concentrations between 0.1 and 10.0 g/L, were used for calibration. Results were expressed as means ± standard deviations (S.D.) of two independent experiments, each performed with three analytical replicates.

The protein concentration in the different milk samples was determined in triplicate using the Lowry method [25], with bovine serum albumin (Sigma, St. Louis, MO, USA) used to construct a standard curve (0.050–0.500 g/L) relating protein concentration to absorbance at 750 nm. Since lactose can interfere with protein determination in milk [26], the contribution of a lactose solution at 50 g/L (equivalent to that in UHT whole milk) to the measured protein content was quantified using the Lowry method [25]. The results showed that this lactose concentration produced an apparent protein concentration of 4.0 g/L, corresponding to an overestimation of 0.08 g of protein per gram of lactose. Therefore, UHT whole milk samples were diluted 100-fold with sterile distilled water to reduce lactose interference to 0.04 g of protein per liter of milk during protein determination using the Lowry method [25]. This dilution step also ensures that the protein concentration falls within the range of the bovine serum albumin standards (0.050–0.500 mg/L) used to construct the calibration curve relating protein concentration to absorbance at 750 nm. Using this approach, the initial protein content of the commercial UHT whole milk determined by the Lowry method was consistent with the manufacturer’s specifications (3.1 g/100 mL).

To determine mineral content, triplicate samples (2 g) of unfermented milk and kefir were digested with 1 mL of H_2_O_2_ (35 wt% solution; AnalytiChem Belgium NV, Zedelgem, Belgium) and 6 mL of HNO_3_ (Trace Metal grade; Fisher Scientific S.L., Madrid, Spain). Analytical blanks were prepared in the same manner. Digestion was performed using a Mars 6 microwave digester (CEM Corporation, Charlotte, NC, USA) at 1000 W, with a 15 min ramp to 195 °C followed by a 20 min hold at 195 °C. The digested samples were diluted to 50 mL with ultrapure water. Blank samples contained 1 mL H_2_O_2_ and 6 mL HNO_3_ and were subjected to the same digestion procedure as the unfermented and fermented milk samples [27].

Mineral concentrations were determined using an Agilent 5800 ICP–OES optical emission spectrometer with rhodium (AnalytiChem Canada Inc., New York, NY, USA) as the internal standard, according to the manufacturer’s instructions (Agilent Technologies, Inc., 2019, Mulgrave, Australia). A multi-element calibration solution containing 27 elements (100 µg/mL each of Al, Sb, As, Ba, Be, B, Cd, Ca, Cr, Co, Cu, Fe, Pb, Mg, Mn, Mo, Ni, K, Se, Si, Ag, Sr, Na, Tl, Ti, V, and Zn in 5% HNO_3_, trace HF; reference 19006500.L1, CPAchem Ltd., Bogomilovo, Bulgaria) was used to construct the calibration curves. The R^2^ values obtained for all calibration curves were higher than 0.9999 with recoveries percentages (between 92% and 108%) within the range typically reported for elemental analysis in food matrices [28]. The equipment was configured with an Agilent SPS 4 autosampler. The sample introduction system consisted of a Seaspray glass concentric nebulizer, a double pass cyclonic spray chamber and an easy-fit torch one piece 5100 DV.

The phosphorus concentration was determined according to the method described by Gliszczyńska-Świgło and Rybicka [29].

For the determination of vitamin content, samples were prepared in triplicate by mixing with 50% acetonitrile in acidified water, followed by vigorous shaking for 1 min and roller-mixing for 10 min (protected from light). After centrifugation (10,000× *g* for 10 min), the supernatant was filtered through hydrophilic polytetrafluoroethylene syringe filters (0.2 μm pore size; Fisher Scientific S.L., Madrid, Spain). A composite standard solution was prepared daily by mixing and diluting individual vitamin stock solutions in Milli-Q water to obtain a final concentration of 1000 ppb for each vitamin [30].

The vitamin content (thiamine [B1], riboflavin [B2], nicotinic acid [B3], pantothenic acid [B5], pyridoxal 5′-phosphate [active coenzyme form of vitamin B6], pyridoxal [aldehyde form of vitamin B6], pyridoxine [alcohol form of vitamin B6], pyridoxamine [B6], biotin [B7], cobalamin [B12], ascorbic acid [C], ergocalciferol [D2], and cholecalciferol [D3]) in unfermented and fermented milk samples was determined by HPLC–tandem mass spectrometry. The system consisted of an Agilent 1260 Series HPLC (Palo Alto, CA, USA) coupled to a SCIEX Triple Quad 3500 equipped with an electrospray ionization (ESI) source. Chromatographic separation was achieved using a Luna C18 column (150 mm × 2 mm internal diameter, 3 μm particle size) from Phenomenex (Madrid, Spain).

The mobile phase consisted of (A) water with 0.1% formic acid (Sigma-Aldrich, Alcobendas, Madrid, Spain) and (B) acetonitrile (Merck, Darmstadt, Germany) with 0.1% formic acid, using the following gradient: 0–2 min, 100% A; 2–7 min, 35% A; 7–8 min, 0% A; and 8.1–15.0 min, 100% A. The flow rate was 400 µL/min, and the column temperature was maintained at 40 °C. Nitrogen was used as both the nebulizer and collision gas. Vitamins were detected in a single LC–MS/MS run following the methodology described by Gentili et al. [30].

The limits of detection (LODs) and quantification (LOQs) for the qualifier transitions of water-soluble vitamins in selected food matrices (maize flour, green kiwi, golden kiwi, and tomato pulp) were reported by Gentili et al. [30]. The LODs for vitamins B1, B2, B3, B5, pyridoxal 5′-phosphate, pyridoxal, pyridoxine, pyridoxamine, B7, B12, and C ranged from 2.0 to 12.9, 4.0 to 6.2, 10.5 to 27.5, 10.0 to 31.5, 19.2 to 44.0, 14.4 to 43.0, 0.9 to 11.0, 0.68 to 1.6, 5.2 to 16.5, 6.6 to 8.0, and 546 to 30,216 ng/g, respectively [30]. The corresponding LOQs were 6.0 to 38.7, 12.0 to 18.6, 31.5 to 82.5, 30.0 to 70.5, 57.6 to 132.0, 43.2 to 129.0, 2.7 to 33.0, 2.04 to 4.8, 15.6 to 49.5, 19.8 to 24.0, and 1638 to 90,648 ng/g, respectively, for the same vitamins [30]. Additionally, the reported LOD values for vitamins D2 and D3 in infant milk formula were 2.0 and 4.7 ng/g, respectively, while the corresponding LOQs were 6.1 and 14.4 ng/g, respectively [31].

### 2.5. Volatile Composition of Substrate and Fermented Beverages

VOCs were determined in triplicate following the procedure described by Bazán et al. [23], using 3-octanol (50.32 mg/L in absolute ethanol) as an internal standard.

The sensory relevance of these compounds was evaluated through odor activity values (OAVs), defined as the ratio between compound concentration and its odor perception threshold (OPT). Compounds with an OAV equal to or greater than 1.0 are considered to make a direct contribution to the overall aroma of the beverage [23].

### 2.6. Antibacterial Activity Assay

The total antibacterial activity produced by MKG and WKG in whole milk was quantified in aliquot B using a photometric method, as described by Cabo et al. [32]. Samples of fermented whole milk were collected at different time points and acidified to pH 3.5 with 5 N HCl (Sigma-Aldrich, Alcobendas, Madrid, Spain) to extract bacteriocin molecules adsorbed onto the cell walls of the producing strains. The samples were prepared in triplicate and then heated in a boiling water bath for 3 min to inactivate the cells. After cooling to room temperature, the samples were centrifuged at 27,200× *g* for 15 min at 4 °C [32]. The pellets were discarded, and the supernatants were adjusted to pH 6.0 with 5 N NaOH (Sigma-Aldrich) and appropriately diluted in sterile distilled water to obtain a range of dilutions.

Equal-volume aliquots of triplicate diluted samples and a 12 h culture of *Carnobacterium piscicola* CECT 4020 (Spanish Type Culture Collection, Valencia, Spain), used as the indicator strain and previously adjusted to an absorbance of 0.2 in MRS broth buffered (pH 6.3) with 0.05 M sodium biphthalate–NaOH, were combined in culture tubes and incubated for 6 h at 30 °C and 200 rpm. Controls consisted of triplicate supernatants obtained from centrifuged milk (27,200× *g* for 15 min at 4 °C), adjusted to pH 6.0, and incubated with the same indicator culture under identical conditions. After measuring the absorbance of each sample (700 nm), the inhibition index (II) was calculated as follows [32]:

$$II=1-\frac{SA}{CA}$$

where SA and CA represent the mean absorbance values of the samples and controls, respectively. Dose–response curves (II versus the inverse of each dilution) were constructed, and the inhibitory dose 50 (ID_50_) for each sample was determined by fitting a dose–response model to the experimental data [33]. TAA was expressed as antibacterial activity units (AUs) per milliliter of sample, with the AU defined as the ID_50_ obtained from the corresponding dose–response curve [33].

### 2.7. Statistical Analyses

All fermentations were carried out in duplicate (independent runs), and each fermentation type (MKG and WKG) was analyzed separately in triplicate (analytical replicates). Accordingly, the total number of observations for each kefir grain fermentation at each sampling time was 2 runs × 3 replicates, resulting in 6 observations. Data are presented as mean ± S.D., as specified in the figure legends.

Mean values of culture pH; counts (expressed as log CFU/mL) of LAB, AAB, yeasts, Enterobacteriaceae, and *Pseudomonas* spp.; concentrations of proteins, lactose, lactic acid, acetic acid, ethanol, and glycerol; vitamins; minerals; volatile compounds; and TAA levels in unfermented and fermented milk were compared statistically using one-way analysis of variance, with a significance threshold of 5%. Games–Howell or Tukey post hoc tests were applied when variances were heterogeneous or homogeneous, respectively, following assessment of variance homogeneity using Levene’s test.

The logistic increase in selected culture variables (e.g., lactic acid, acetic acid, ethanol, and TAA) was described using the generalized logistic Equation (1) [34]:

(1) $$CV(t)=\frac{{CV}_{max}}{1+a\cdot e^{\left(-b\cdot t-c\cdot t^{2}\right)}}$$

where *CV_max_* is the maximum value of the culture variable; *a*, *b*, and *c* are constants; and *t* represents time.

In the case of logistic decrease (e.g., pH, lactose, and proteins), the positive sign of the constant *a* in Equation (1) was replaced by a negative sign, yielding Equation (2):

(2) $$CV(t)=\frac{{CV}_{min}}{1-a\cdot e^{\left(-b\cdot t-c\cdot t^{2}\right)}}$$

where *CV_min_* is the minimum value of the culture variable, and *a*, *b*, *c*, and *t* have the same meanings as in Equation (1).

Models fitting was performed by minimizing the sum of squared differences between observed and predicted values using a nonlinear least-squares (quasi-Newton) method implemented with the Solver add-in in Microsoft Excel 2007 (Microsoft, Redmond, WA, USA).

Principal component analysis (PCA) was applied to evaluate the relationships among the five beverages (UWM, MKG-24 h, MKG-48 h, WKG-24 h, and WKG-48 h) and to reduce the dimensionality of the dataset into principal components while preserving the majority of the variability contained in the original dataset. The variables included in the analysis were pH; microbiological counts (LAB, AAB, and yeasts); concentrations of lactose; proteins; metabolites (organic acids and alcohols); minerals; vitamins; the number of volatile compounds with OAV ≥ 1.0; and TAA. Bartlett’s test of sphericity was applied to assess the suitability of the data for PCA, and factors with eigenvalues greater than 1.0 were retained according to the Kaiser criterion [23].

Cluster analysis was performed to assess the similarities and differences among unfermented milk and the four fermented beverages produced from this substrate using MKG and WKG at two fermentation times (24 and 48 h). The analysis was based on the principal factor scores obtained from the PCA. Euclidean distance was used as the similarity metric, and clustering was carried out using the nearest neighbor linkage method (single linkage).

Statistical comparisons, principal component analysis (using the Factor Analysis module), and cluster analysis (using the Cluster Analysis module) were performed using IBM^®^ SPSS^®^ Statistics for Windows (Version 25.0; IBM Corp., Armonk, NY, USA).

## 3. Results and Discussion

### 3.1. Fermentation Kinetics of Whole Milk with MKG and WKG

The viable cell counts of LAB, AAB, and yeasts in MKG were 9.2 ± 0.6 × 10^7^, 6.5 ± 0.8 × 10^6^, and 8.5 ± 0.8 × 10^7^ CFU/g, respectively. In WKG, the counts of LAB, AAB, and yeasts were 6.7 ± 0.7 × 10^6^ CFU/g, 6.0 ± 0.5 × 10^6^ CFU/g, and 1.8 ± 0.2 × 10^6^ CFU/g, respectively. As observed, the viable cell counts of LAB and yeasts in MKGs are approximately one order of magnitude higher than those in WKGs, while AAB counts were similar between the kefir grains. This difference could influence the time course of the various culture variables during WKG and MKG fermentations.

**Figure 1:**
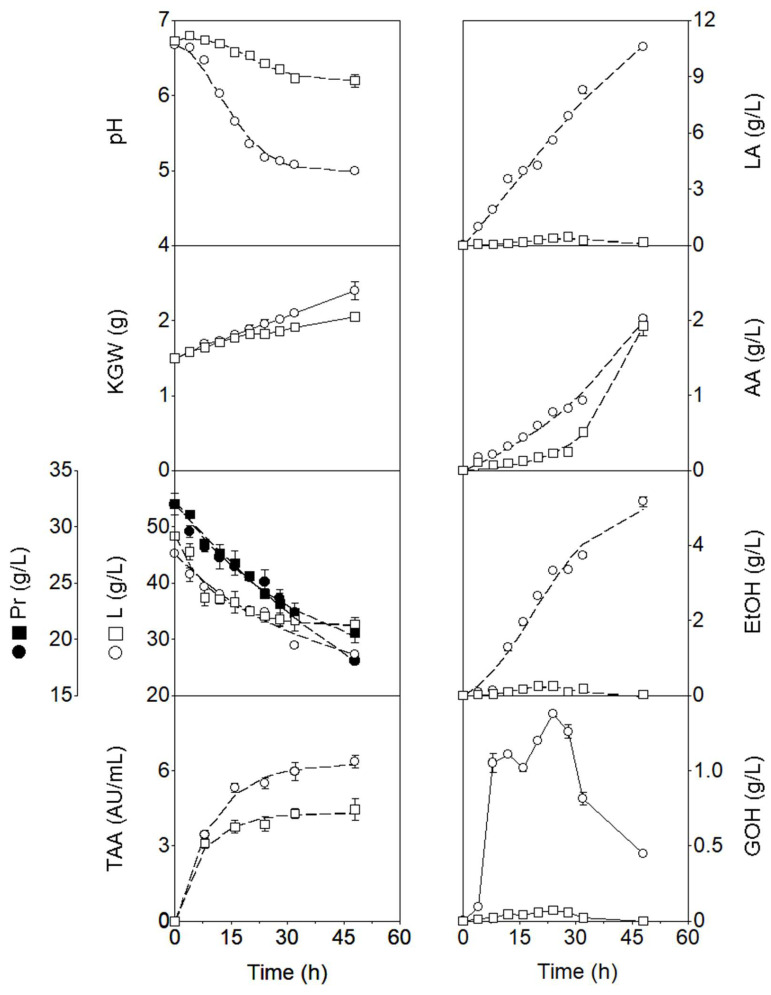
Kinetics of pH, lactic acid (LA), kefir grain weight (KGW), acetic acid (AA), lactose (L), protein (Pr), ethanol (EtOH), glycerol (GOH), and total antibacterial activity (TAA) during 48 h of fermentation of UHT whole milk with MKG (circles) and WKG (squares). The dashed lines fitted to the experimental LA, AA, EtOH, and TAA data were obtained using logistic model (1), whereas those fitted to the experimental pH, L, and Pr data were obtained using logistic model (2). Data points represent means ± S.D. from two experiments with three replicates each.

The 48-h evolution of cultures inoculated with both kefir grains in whole milk is shown in Figure 1. From a kinetic perspective, the pH profile during MKG fermentation exhibited a logistic decrease (see dashed lines fitted to the experimental pH data) from the onset of incubation (6.70 ± 0.03) to 4.99 ± 0.01 at the end of incubation, in parallel with a logistic increase in lactic acid and acetic acid production (see dashed lines fitted to the corresponding experimental data), reaching final concentrations of 10.58 ± 0.00 g/L and 2.02 ± 0.02 g/L, respectively.

Different concentrations of lactic acid and acetic acid have been reported by various researchers during the fermentation of different types of cow’s milk with MKG. For example, Magalhães et al. [35] reported lactic acid and acetic acid concentrations of 17.40 g/L and 2.73 g/L, respectively, after 24 h of fermentation of pasteurized cow’s milk inoculated with Brazilian MKG (0.011%, *w*/*v*).

Gamba et al. [36] reported lower concentrations of lactic acid (13.06 g/L) and acetic acid (1.35 g/L) after 24 h of cow’s milk fermentation, even though they used a higher MKG inoculum (10%, *w*/*v*) than that used by Magalhães et al. [35]. However, an even lower lactic acid concentration (6.30 g/L) was obtained by Alves et al. [37] in UHT semi-skimmed cow’s milk using the same inoculum concentration (10%, *w*/*v*) of MKG for 24 h.

Leite et al. [38] detected lower concentrations of lactic acid (7.38 g/L) and acetic acid (0.93 g/L) during 24 h of fermentation of commercial UHT skimmed cow’s milk with 3% (*w*/*v*) MKG. In contrast, Fiorda et al. [20] reported a considerably higher lactic acid concentration (30.45 g/L) after 24 h of cow’s milk fermentation using an inoculum size of 5% (*w*/*v*) MKG. Interestingly, a lactic acid concentration of 10.73 g/L was reported after 22 h of fermentation of heat-treated cow’s milk using a lower inoculum level (2%, *w*/*v*, MKG) [39] than that used by Alves et al. [37].

Unfortunately, acetic acid concentrations were not reported in the studies by Fiorda et al. [20], Alves et al. [37], or Kök-Tas et al. [39].

During the initial phase of WKG fermentation, the pH showed a slight increase within the first 4 h of incubation, rising from 6.70 ± 0.03 to 6.80 ± 0.02, followed by a gradual decline to 6.78 ± 0.03 at 8 h. This trend corresponded to a decrease in lactic acid concentration from 0.28 ± 0.00 g/L at inoculation to 0.07 ± 0.01 g/L at 4 h and 0.04 ± 0.00 g/L at 8 h. Meanwhile, acetic acid concentration increased slightly from 0.09 ± 0.02 g/L at inoculation to 0.11 ± 0.03 g/L at 4 h, before decreasing to 0.07 ± 0.00 g/L at 8 h.

Between 8 h and 28 h of incubation, the pH decreased to 6.35 ± 0.04 at a rate of 0.02 h^−1^, consistent with the increase in lactic acid and acetic acid concentrations to 0.46 ± 0.18 g/L and 0.25 ± 0.01 g/L, respectively (Figure 1). Subsequently, the pH declined further to 6.23 ± 0.08 at 32 h (at a rate of 0.03 h^−1^) and finally to 6.20 ± 0.04 at 48 h (at a rate of 1.87 × 10^−3^ h^−1^). This later decrease was mainly associated with the rise in acetic acid concentration to 0.51 ± 0.06 g/L at 32 h and 1.93 ± 0.13 g/L at 48 h. In contrast, lactic acid concentrations decreased to 0.26 ± 0.08 g/L at 32 h and 0.18 ± 0.04 g/L at 48 h.

The reduction in lactic acid concentration during fermentation is commonly attributed to its consumption by lactic acid-assimilating yeasts (such as *Torulaspora delbrueckii*, *Debaryomyces hansenii*, *Candida guilliermondii*, and *Saccharomyces cerevisiae*) [40,41,42,43], as well as by certain *Lactobacillus* species (e.g., *L. buchneri* and *L. parabuchneri*) [44,45,46] present in kefir grains.

The smaller overall pH decrease observed in the WKG culture is consistent with the significantly lower (*p* < 0.05) concentration of lactic acid in this fermentation compared with the MKG culture. In contrast, the similar (*p* > 0.05) acetic acid production in MKG (2.02 ± 0.02 g/L) and WKG (1.93 ± 0.13 g/L) cultures does not explain the difference in pH reduction observed between the two fermentations.

LAB and AAB present in kefir grains are the microorganisms responsible for the production of organic acids in kefir beverages, with lactic acid production commonly associated with the homofermentative metabolism of sugars by LAB [16,47,48]. However, heterofermentative LAB also contribute to lactic acid production and are capable of producing acetic acid [49,50] and ethanol [50]. In addition, acetic acid is mainly produced by AAB, which can synthesize this organic acid through the assimilation of sugars (glucose and fructose), alcohols (ethanol and glycerol), or lactic acid [51,52].

Regarding alcohol production, the final concentrations of ethanol and glycerol, mainly attributed to the metabolic activity of yeasts [52], in the MKG culture were consistently higher than those observed in the WKG culture (Figure 1). From a kinetic perspective, however, the evolution of these two alcohols differed between fermentations.

In the MKG culture, ethanol concentrations increased throughout the 48 h incubation period, following a sigmoidal (logistic) profile and reaching a final value of 5.17 ± 0.13 g/L. In contrast, glycerol levels increased to 1.10 ± 0.01 g/L at 12 h, decreased slightly to 1.02 ± 0.02 g/L at 16 h, then increased linearly to 1.37 ± 0.01 g/L at 24 h, and finally decreased in a sigmoidal manner to 0.45 ± 0.00 g/L at 48 h.

In the WKG fermentation, ethanol accumulated in the fermented substrate up to 0.26 ± 0.00 g/L at 20 h and then decreased to 0.03 ± 0.00 g/L at 48 h. Similarly, glycerol concentration increased to 0.07 ± 0.00 g/L at 24 h and subsequently decreased in a sigmoidal manner until it was no longer detectable at the end of the incubation period. The decrease in the concentrations of both alcohols in the fermentation substrate has commonly been attributed to their assimilation by AAB [6,51,52] or their oxidation into organic acids [53].

Lower ethanol concentrations have been reported in other studies, including 0.50 g/L [35], 0.32 g/L [1], 0.11 g/L [39], and 0.02 g/L [36] in cow’s milk kefirs, as well as approximately 31.80 mg/L in buffalo and cow’s milk kefirs [54] produced with MKG. Interestingly, Alves et al. [37] did not detect ethanol production in cow’s milk kefir. However, other studies have reported higher ethanol levels, such as 2.68 g/L [55] and 3.54 g/L [20] in kefir beverages produced with MKG, which fall within the range obtained in the present MKG fermentation: 3.33 ± 0.00 g/L at 24 h and 5.17 ± 0.13 g/L at 48 h (Figure 1).

Overall, these findings indicate that the production of organic acids and ethanol in kefir beverages is influenced by the type of milk and by the type and concentration of kefir grains used as inoculum, as well as by the number and abundance of microbial strains present in them. In addition, the concentrations of these metabolites in kefir beverages result from their simultaneous production and consumption by the complex microflora present in kefir grains, which likely prevents the accumulation of inhibitory levels for LAB, AAB, and yeasts [23,50]. However, studies using advanced molecular techniques to identify the strains present in the grains, followed by their isolation and the development of pure and mixed cultures, could help clarify their contribution to metabolite production.

Comparison of the levels of organic acids and alcohols produced by WKGs in whole milk with those reported in the literature is challenging, as these grains have not previously been used for milk fermentation. Therefore, the concentrations of lactic acid, acetic acid, and ethanol obtained in the present WKG milk culture (Figure 1) were compared with those reported for non-dairy kefir-like beverages.

For example, Magalhães et al. [56] reported lactic acid, acetic acid, and ethanol concentrations of 1.82, 1.40, and 0.84 g/L, respectively, in a kefir-like beverage obtained from a sugary substrate (5% brown sugar in distilled water) fermented with 0.011% (*w*/*v*) WKG for 24 h. These values are markedly higher than those observed in the present WKG milk fermentation at 24 h (0.39 g/L lactic acid, 0.23 g/L acetic acid, and 0.25 g/L ethanol). From a kinetic perspective, a reduction in lactic acid concentration was observed in both studies; however, in the sugary beverage the decline occurred after 12 h of fermentation, whereas in the present WKG milk culture (Figure 1) it was observed between 32 and 48 h. In the sugary substrate, acetic acid and ethanol concentrations increased up to 12 h and remained nearly constant thereafter [56].

In kefir-like beverages produced by fermenting apple, dragon fruit, kiwifruit, and orange juices with 0.1% (*w*/*w*) WKG for 48 h, lactic acid, acetic acid, and ethanol concentrations ranged from 0.26 to 3.14 g/L, 0.10 to 1.61 g/L, and 0.30 to 1.77 g/L, respectively, after 24 h of incubation [57]. At 48 h, these ranges broadened to 0.30–9.03 g/L (lactic acid), 0.19–1.77 g/L (acetic acid), and 0.32–5.60 g/L (ethanol). In comparison, the concentrations obtained in the present WKG milk fermentation were substantially lower for lactic acid (0.39 and 0.18 g/L at 24 and 48 h, respectively) and ethanol (0.25 and 0.03 g/L), while acetic acid levels (0.23 and 1.92 g/L) were within or slightly above the upper range reported for fruit-based beverages at 48 h.

Kefir grain weight increased linearly in the MKG culture and showed an almost linear trend in the WKG culture (Figure 1), likely due to the proliferation of grain-associated microbiota and the synthesis of kefiran. The production of this exopolysaccharide has been attributed to several *Lactobacillus* strains, including *L. kefiranofaciens*, *L. kefir*, *L. kefirgranum*, *L. parakefir*, *L. delbrueckii* subsp. *bulgaricus*, and *L. plantarum* [22,42,58,59].

Lactose concentrations in both MKG and WKG cultures decreased throughout the incubation period (Figure 1); however, the final levels (27.25 ± 0.37 g/L and 32.65 ± 1.19 g/L, respectively) differed significantly (*p* < 0.05). Accordingly, lactose consumption in each fermentation was also significantly different (*p* < 0.05), reaching 18.04 ± 0.33 g/L (39.83%) in the MKG culture and 15.73 ± 0.93 g/L (32.52%) in the WKG culture.

A high variability in lactose consumption has been observed by various researchers during milk fermentation with MKG. For example, lactose consumptions of 15.65 g/L (33.03%) and 16.00 g/L (33.06%) were reported after 24 h of fermentation of pasteurized whole milk with Brazilian kefir grains (0.011%, *w*/*v*) [35] and commercial UHT skimmed milk with 3% (*w*/*v*) MKGs [1], respectively.

However, Irigoyen et al. [60] observed considerably lower lactose consumption (20–25%) after 24 h of fermentation of full-fat UHT cow’s milk with 1% or 5% (*w*/*w*) MKG, respectively. Similarly, Gamba et al. [36] reported a 22.13% reduction in the initial lactose concentration after 24 h of cow’s milk fermentation, while Alves et al. [37] observed an even lower lactose consumption (13.50%).

Protein concentrations showed an almost linear decrease in both cultures, resulting in significantly different (*p* < 0.05) protein concentrations of 25.09 ± 0.00 g/L and 24.04 ± 0.42 g/L at 24 h of incubation, and 18.11 ± 0.37 g/L and 20.55 ± 0.88 g/L at 48 h of incubation in the MKG and WKG cultures, respectively (Figure 1). The corresponding final protein consumption percentages were 21.59% and 24.87% (at 24 h) and 43.41% and 33.92% (at 48 h), respectively. The final protein concentrations in the fermented milks at 24 h and 48 h, resulting from their assimilation by the microbial populations of the kefir grains, are considerably lower than the values above 31.00 g/L reported for milk kefir by Rutkowska et al. [9] and Otles and Çağındı [12]. Unfortunately, these researchers did not report the initial protein concentrations of the milks used as substrates; therefore, calculation of the protein consumption percentages was not possible.

In contrast, the protein consumption observed in the MKG and WKG cultures was within the range reported by Arroum et al. [61] in kefir samples produced by fermenting camel milk kefir (37.82 ± 0.37 g protein/L) with 2%, 5%, and 10% MKG (*w*/*v*) for 18 h. These researchers reported final protein levels of 22.04 ± 1.30, 25.34 ± 3.60, and 31.10 g/L for fermentations with 2%, 5%, and 10% MKG, respectively, corresponding to protein consumptions of 41.72%, 33.00%, and 17.77%.

Interestingly, fermentation resulted in an increase in protein concentration in kefir samples produced from milks of Saanen goats with intensive feeding, Saanen goats with extensive feeding, Hair goats with extensive feeding, and Holstein cows with intensive feeding [62]. The corresponding milks contained 3.61 ± 0.17, 3.42 ± 0.10, 4.79 ± 0.38, and 3.37 ± 0.27 g protein/100 g milk, respectively. The protein concentrations in the corresponding kefir samples were 4.00 ± 0.36, 3.69 ± 0.14, 5.21 ± 0.08, and 3.57 ± 0.09 g protein/100 g kefir. This increase in protein concentration in the kefir samples compared with the unfermented substrate is related to the coagulation of milk proteins, caused by the metabolic activity of the LAB present in the kefir grains [11].

On the other hand, TAA in both fermentations increased in a logistic manner (see dashed lines fitted to the experimental TAA data), reaching final levels of 6.36 ± 0.26 AU/mL and 4.46 ± 0.42 AU/mL in the MKG and WKG cultures, respectively (Figure 1). This significant difference (*p* < 0.05) in final TAA values may be attributed to the higher production of organic acids and alcohols (Figure 1), and possibly bacteriocins produced by the LAB present in kefir grains [23] in the MKG culture compared with the WKG fermentation.

### 3.2. Growth Kinetics of Viable LAB, AAB, and Yeasts in MKG and WKG Fermentations

**Figure 2:**
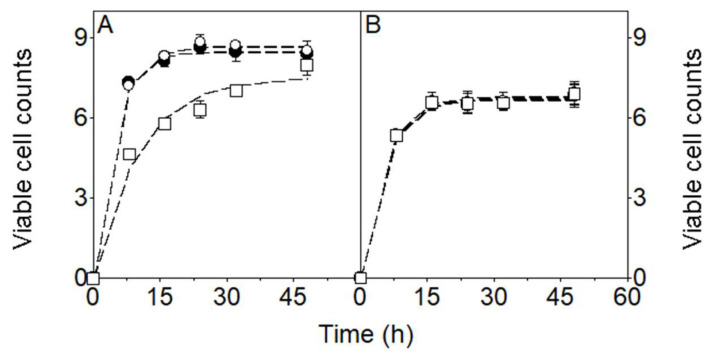
Viable cell counts (log CFU/mL) of LAB (closed circles), AAB (open circles), and yeasts (open squares) during 48 h of fermentation of UHT whole milk with MKG (**A**) and WKG (**B**). The dashed lines, fitted to the experimental growth data, were obtained using the logistic model (1). Data points represent means ± S.D. from two independent experiments, each performed in triplicate.

As shown in Figure 2, the counts of the three microbial groups in the MKG culture increased in a sigmoidal (logistic) manner (see dashed lines fitted to the experimental viable cell count data). LAB and AAB populations evolved in parallel, reaching their highest mean counts at 24 h of incubation, with similar values (*p* > 0.05) of 8.64 log CFU/mL for LAB and 8.83 log CFU/mL for AAB. Subsequently, both populations decreased but not significantly (*p* > 0.05) to mean values of 8.40 log CFU/mL (LAB) and 8.52 log CFU/mL (AAB) at 48 h of incubation (Figure 2A). In contrast, yeast populations increased more slowly, reaching significantly different (*p* < 0.05) mean counts of 6.32 log CFU/mL at 24 h and 8.00 log CFU/mL at 48 h. The increase in yeast counts from 24 to 48 h is practically relevant, as it led to increased ethanol production (Figure 1), which may affect product acceptability, as well as enhanced formation of volatile aroma compounds, thereby contributing to a more complex flavor profile in kefir beverages.

The viable counts obtained in the MKG culture were within the ranges reported for other kefir beverages for the three microbial groups. For example, Gamba et al. [36] reported comparable LAB (8.0–9.0 log CFU/mL) and yeast counts (6.0–7.0 log CFU/mL) in cow’s milk kefir, although AAB counts (6.0–7.0 log CFU/mL) were lower than those observed after 24 h in the present MKG fermentation.

Irigoyen et al. [60] obtained comparable LAB counts—lactobacilli (8.20 and 8.00 log CFU/mL) and lactococci (8.40 and 8.20 log CFU/mL)—in milk kefirs produced with 1% or 5% (*w*/*w*) MKG. However, lower yeast (5.40 and 5.80 log CFU/mL) and AAB (6.00 and 6.30 log CFU/mL) counts were reported in those cultures.

Magalhães et al. [35] reported considerably higher LAB levels (12.41 log CFU/mL), while AAB and yeasts reached viable counts of 7.72 and 8.11 log CFU/mL, respectively, in whole milk kefir. Additionally, Alves et al. [37] reported final LAB and yeast counts of 7.84 and 6.30 log CFU/mL, respectively.

In the WKG culture (Figure 2B), the three microbial populations exhibited similar growth profiles, reaching comparable mean counts (*p* > 0.05) at 24 h (6.49 log CFU/mL for LAB, 6.60 log CFU/mL for AAB, and 6.54 log CFU/mL for yeasts). At the end of incubation, final counts (*p* > 0.05) were 6.84 log CFU/mL (LAB), 6.95 log CFU/mL (AAB), and 6.89 log CFU/mL (yeasts).

As no studies were found addressing milk fermentation with WKGs, these results (Figure 2B) were compared with microbial loads reported for kefir-like beverages produced from nondairy substrates using WKGs. For example, Magalhães et al. [56] reported final counts of 8.41 log CFU/mL (*Lactococcus*), 8.32 log CFU/mL (*Lactobacillus*), 8.31 log CFU/mL (*Acetobacter*), and 7.31 log CFU/mL (yeasts), which are generally higher than the values observed in the present WKG milk fermentation.

Similarly, Dikmetas et al. [57] observed the highest *Lactobacillus* counts in a dragon fruit kefir-like beverage at 24 h (6.67 log CFU/mL) and 48 h (7.78 log CFU/mL), while the highest *Lactococcus* counts were detected in the apple beverage at 24 h (6.40 log CFU/mL) and in the kiwifruit beverage at 48 h (7.49 log CFU/mL). Yeast populations reached their highest levels in the kiwifruit beverage at 24 h (6.80 log CFU/mL) and in the dragon fruit beverage at 48 h (8.57 log CFU/mL). Compared with these nondairy substrates, the microbial counts obtained in the present WKG milk fermentation (Figure 2B) were generally lower than those reported for sugar-based media but within the range described for fruit-based kefir-like beverages, particularly for LAB and yeasts after 48 h of incubation.

However, in both MKG and WKG beverages, the average viable counts of LAB and yeasts—microorganisms with reported probiotic properties [24,63,64]—at fermentation times commonly used for milk kefir production (24 or 48 h) were within the range of 10^6^ to 10^7^ CFU/mL. The FAO/WHO document [65] provides a definition of probiotics and outlines guidelines for their evaluation, including assessment methods, safety considerations, product specifications, quality assurance, regulatory aspects, and post-market surveillance. However, it does not establish a universal quantitative threshold [65,66]. Nevertheless, the range of 10^6^–10^7^ CFU/mL is widely accepted in the literature as the minimum effective level derived from these guidelines [67,68,69].

Additionally, the counts of *Pseudomonas* and Enterobacteriaceae in the MKG and WKG samples were well below the detection limit (30–300 CFU/mL), indicating that these samples had good hygienic quality and were safe for human consumption.

### 3.3. Vitamin Contents in MKG and WKG Beverages

The concentrations of B-group vitamins and vitamin D3 in unfermented whole milk (UWM) and in samples fermented with MKG and WKG for 24 and 48 h are presented in Table 1.

The concentrations of vitamins in UWM and in milk fermented with MKG and WKG for 24 and 48 h showed clear variations (Table 1). As previously indicated [70,71,72,73,74,75], the vitamin content can vary among dairy products as a result of processing conditions, as well as the consumption or synthesis of vitamins by the microbiota of kefir grains during the fermentation of dairy substrates.

Riboflavin (B2) concentrations increased during fermentation with MKG, rising from 239.65 µg/L in UWM to 262.28 µg/L at 24 h (not significant, *p* > 0.05) and 277.55 µg/L at 48 h (*p* < 0.05), corresponding to an increase of approximately 16% after 48 h, consistent with observations by LeBlanc et al. [70,71] and Linares et al. [72]. In contrast, WKG fermentation yielded lower B2 levels than the original milk, with 206.28 µg/L at 24 h (*p* > 0.05) and 215.40 µg/L at 48 h (*p* < 0.05), suggesting partial microbial consumption or limited biosynthesis of this vitamin in the WKG culture. These results indicate that the microbiota present in MKG may have a greater capacity for riboflavin synthesis than that present in WKG.

A marked increase (*p* < 0.05) was observed for nicotinic acid (B3) in both fermentations. Concentrations increased from 116.64 µg/L in UWM to 352.67 µg/L in MKG-48 h and 347.55 µg/L in WKG-48 h (Table 1), representing approximately a threefold increase. This strong accumulation suggests active microbial production of B3 during kefir fermentation, which is consistent with the known metabolic capabilities of the microbiota present in the kefir grains. These results are consistent with those reported by LeBlanc et al. [71], who described LAB-mediated production of B3 in fermented dairy products.

Similarly, pantothenic acid (B5) increased in all fermented samples compared with UWM (1231.75 µg/L). The highest concentrations (*p* < 0.05) were observed in MKG at 24 h (1735.65 µg/L) and 48 h (1746.19 µg/L), while slightly lower values were detected in WKG samples (1589.25 µg/L at 24 h (*p* < 0.05) and 1519.50 µg/L at 48 h (*p* > 0.05)). These results suggest that fermentation enhances B_5_ levels, particularly in MKG cultures.

The behavior of vitamin B6 vitamers was more complex. Pyridoxal concentrations did not exhibit a significant decrease (*p* > 0.05) in MKG (20.25 µg/L) and WKG (22.33 µg/L) beverages after 24 h of incubation, compared with 24.79 µg/L in UWM. However, a substantial decrease (*p* < 0.05) was observed after prolonged fermentation, reaching 10.37 µg/L in MKG and 14.63 µg/L in WKG at 48 h, suggesting microbial utilization or conversion. In contrast, pyridoxine increased markedly (*p* < 0.05) from 0.12 µg/L in UWM to 4.52 µg/L in MKG-48 h and 3.56 µg/L in WKG-48 h, suggesting microbial transformation of pyridoxal into pyridoxine or de novo synthesis [70,71]. Pyridoxamine showed only minor variations, remaining within a narrow range across samples, especially between the unfermented substrate and the MKG-48 h and WKG-48 h samples (*p* > 0.05).

Biotin (B7) increased during the early stages of fermentation, reaching 8.83 µg/L in MKG at 24 h (*p* < 0.05) and 6.47 µg/L in WKG at 24 h (*p* > 0.05), but decreased slightly after prolonged fermentation compared with 24 h (*p* < 0.05), particularly in WKG at 48 h (5.56 µg/L), indicating possible microbial consumption following initial production [70,71,72,73,74,75].

In contrast to the B vitamins, cholecalciferol (vitamin D3) decreased progressively (*p* < 0.05) during fermentation. Concentrations declined from 162.70 µg/L in UWM to 111.38 µg/L in MKG at 48 h and 127.50 µg/L in WKG at 48 h, suggesting either microbial degradation or adsorption to the growing biomass during fermentation [70,71,72,73,74,75].

Overall, fermentation of UHT whole milk with kefir grains significantly modified the vitamin profile of the beverages. MKG generally promoted higher accumulation of B2 and B5, while both MKG and WKG markedly increased B3 and pyridoxine levels. Conversely, vitamin D3 and pyridoxal decreased during fermentation, highlighting the complex metabolic interactions occurring within the kefir microbial consortium. These findings suggest that kefir fermentation can improve the nutritional value of milk by increasing the content of certain B-group vitamins, depending on the type of kefir grains used and the fermentation time. However, additional control experiments are needed to confirm the causes for the variations observed among vitamins during fermentation.

### 3.4. Mineral Content in MKG and WKG Beverages

The mineral levels detected in the fermented samples after 24 and 48 h of fermentation are presented in Table 2. In all samples, K, Ca, P, Na, and Mg were the predominant minerals, whereas Zn, Fe, and Se were present at much lower concentrations, and Mn and Cu were below the detection limit. This distribution is consistent with the typical mineral composition reported for milk and fermented dairy beverages, in which macroelements such as Ca, K, Mg, and Na constitute the major fraction of the mineral profile. Previous studies on kefir and other fermented milk beverages have similarly reported that Ca, K, Mg, and P are the most abundant minerals, while trace elements such as Fe, Cu, Mn, and Zn occur at much lower concentrations [12,27,62,63,76,77].

In this study, comparison of UWM with the fermented samples revealed only moderate, non-significant (*p* > 0.05) variations in mineral concentrations following fermentation with the two kefir grains, indicating that fermentation preserved the mineral nutritional value of the unfermented substrate.

However, studies on kefir composition have reported inconsistent findings, indicating that mineral levels may change during fermentation, with values similar to, higher than, or lower than those of the original milk substrates [6,27,62,63,77,78,79,80].

Trace elements such as zinc, iron, selenium, manganese, and copper are generally present in fermented dairy beverages at low concentrations, depending on the mineral content of the raw milk and environmental factors such as animal diet and soil composition [62]. Previous studies on kefir have also reported low concentrations of iron and selenium, while zinc typically occurs at slightly higher levels due to its association with milk proteins [6,12,27,79].

### 3.5. Volatile Composition of MKG and WKG Beverages

The VOCs present in UWM and in fermented samples obtained after 24 and 48 h of fermentation are presented in Table 3. Thirty-one VOCs, belonging to six chemical families—organic acids, alcohols, ketones, esters, lactones, and other compounds—were identified. Fermentation significantly increased both the diversity and the levels of several VOCs associated with microbial metabolism of lactose, lipids, and amino acids. These changes are driven by the microbial species present in the grains [3,9,11].

The OPT and odor descriptors found in the literature, along with the OAVs of the different VOCs identified in the five beverages are shown in Table 4.

Milk fermented with MKG showed a greater diversity of alcohols and acids, whereas WKG fermentation favored ester formation, especially ethyl esters associated with fruity aromas. Extending fermentation from 24 h to 48 h generally increased the concentration of several compounds, particularly n-decanoic acid, octanoic acid, dodecanoic acid, and lactones, suggesting ongoing lipid hydrolysis and microbial metabolic activity [10,118].

Importantly, the sensory relevance of these compounds depends on their OAVs (Table 4). In this study, the key aroma-active groups in all samples included organic acids (octanoic acid and n-decanoic acid), alcohols (2-nonanol and 2-heptanol), ketones (3-octanone, 2-nonanone, 2-undecanone, and 2-tridecanone), esters (ethyl octanoate and ethyl decanoate), lactones (δ-decalactone, δ-dodecalactone, and γ-dodecalactone), and D-limonene. Collectively, these compounds generate a complex aroma characterized by creamy, coconut-like, fruity, floral, and fatty notes typical of kefir beverages.

#### 3.5.1. Organic Acids

Octanoic acid, n-decanoic acid, and dodecanoic acid were present in all samples, with concentrations increasing during fermentation, particularly after 48 h. Octanoic acid increased from 2.55 mg/L in UWM to 5.69 mg/L in MKG-48 h and 5.23 mg/L in WKG-48 h. Although its OAV was below 1.0 in UWM (0.85), it exceeded the odor threshold after fermentation with MKG and WKG at 48 h, indicating a potential contribution to the aroma of fermented milk (Table 3 and Table 4).

Among the organic acids, n-decanoic acid showed the highest sensory relevance, with OAVs ranging from 2.83 in UWM to 12.74 in WKG-48 h (Table 4). This compound is commonly associated with waxy, fruity, cheesy, and fatty sensory attributes and has also been reported as a key aroma-active component in fermented dairy products [11]. The increase in hexanoic, octanoic [9], and n-decanoic acids during fermentation can be attributed to microbial lipolysis and the subsequent oxidation of milk lipids by kefir microorganisms [11].

Other acids, including hexanoic, pentanoic, and dodecanoic acids, were detected in fermented samples but exhibited OAVs below 1.0, suggesting a minor direct impact on aroma despite their relatively high concentrations (Table 4). Nevertheless, these acids may contribute indirectly to flavor complexity through synergistic interactions with other volatile compounds [17,23].

#### 3.5.2. Alcohols

Alcohols were largely absent in the unfermented milk and were mainly produced during fermentation (Table 3), and their presence in the fermented beverages is associated with the catabolism of carbohydrates and amino acids present in the substrate by the yeasts in kefir grains [9].

Among them, 2-nonanol and 2-heptanol exhibited OAVs greater than 1.0, indicating their contribution to the aroma profile of the fermented drinks. The presence of 2-nonanol, with OAVs ranging from 11.40 to 15.77 across fermented samples, suggests its important role in imparting green and fruity notes to the beverages. Similarly, 2-heptanol displayed OAVs between 3.74 and 8.22, contributing green and fresh aromatic nuances (Table 4).

Phenylethyl alcohol was detected only in MKG-fermented samples and showed an OAV greater than 1.0 (3.02) only at 48 h. This compound is typically produced by yeast metabolism during fermentation [11] and is associated with floral, rosy, flowery, and honey-like aromas (Table 4).

Nerol remained below its odor threshold in the MKG-48 h and WKG-48 h samples, while 2-dodecanol was only detected in WKG-48 h, with an OAV > 1.0 (Table 3 and Table 4).

#### 3.5.3. Ketones

The presence of ketones in fermented beverages is associated with lipolysis, oxidation, and decarboxylation of fatty acids by LAB [122]. These volatile compounds were among the most aroma-active compounds detected in this study, considering their relatively high OAVs (Table 4). In UWM, several ketones exhibited extremely high OAVs, particularly 2-nonanone (440.63), 2-undecanone (428.63), and 2-heptanone (362.38), highlighting their dominant contribution to the typical aroma of the unfermented substrate.

Although the concentrations of some ketones decreased considerably after fermentation, they still exceeded their odor thresholds in most fermented samples (with OAVs remaining well above 1.0) and therefore continued to contribute to the overall aroma. For example, 2-nonanone showed OAVs of 85.56 and 213.00 in MKG-24 h and MKG-48 h, respectively, while 2-undecanone maintained OAVs between 75.60 and 224.60 across fermented samples. These compounds contribute fruity, floral, and slightly musty notes and therefore remain key contributors to the sensory profile of the beverages (Table 4).

3-Octanone also exceeded its odor threshold in all samples, with OAVs ranging from 6.56 to 9.25. This compound is associated with slightly fruity, sweet, and cooked aromas, which may contribute to the overall complexity of the fermented milk (Table 4).

#### 3.5.4. Esters

Esters, commonly associated with fruity and floral aromas, were particularly abundant in WKG-fermented samples, suggesting that the water kefir microbiota promotes enzyme-catalyzed ester formation between alcohols and organic acids [111,112].

Ethyl octanoate was the ester with the highest OAVs in the fermented beverages, especially in WKG samples (240.82 at 24 h and 213.02 at 48 h). This compound is associated with fruity notes reminiscent of apple and banana and is commonly produced by yeast metabolism during fermentation [121,123]. Ethyl decanoate also exhibited OAVs above 1.0 in MKG-48 h and in both WKG samples, contributing fruity and floral characteristics.

In contrast, ethyl hexadecanoate was detected only in WKG-24 h and showed an OAV (1.29) considerably lower than those of ethyl octanoate and ethyl decanoate. Therefore, ethyl hexadecanoate likely has a more limited individual contribution to the flavor of the beverage than the other two esters.

#### 3.5.5. Lactones and Other Compounds

Lactones were among the most potent aroma contributors in all samples, and their increase during fermentation may result from the microbial transformation of hydroxy fatty acids derived from milk lipids [10,118].

δ-Decalactone exhibited extremely high OAVs, increasing from 96.96 in UWM to 616.11 in MKG-48 h. This compound is known for its characteristic coconut-like and creamy aroma and is considered a key flavor component in dairy products [10,88,118,124]. Similarly, δ-dodecalactone and γ-dodecalactone displayed high OAVs across all samples, contributing sweet, creamy, and fruity notes.

Another relevant compound was D-limonene, detected only in MKG and WKG beverages and exhibiting OAVs greater than 1.0, thereby contributing citrus-like and herbal notes to the overall aroma profile [9,121,125].

In any case, the analysis of the aromatic profile of beverages is inherently challenging, since odor perception thresholds and sensory descriptors reported in the literature are often matrix-dependent. These values are typically determined in different experimental systems (e.g., aqueous solutions, model wine systems, dairy matrices, or alcoholic beverages), which can significantly influence volatility, compound–matrix interactions, and therefore perceived aroma intensity (Table 4). This variability complicates direct comparisons across studies and limits the straightforward application of literature-based thresholds to complex fermented beverages. Consequently, interpretation of the aromatic profile must be performed with caution, taking into account the matrix in which both analytical measurements and sensory references were originally obtained (Table 4).

### 3.6. PCA of Unfermented Milk and MKG and WKG Beverages

The results obtained in this study generated a large initial set of variables (30), which made direct comparison among the UWM and the fermented beverage samples (MKG-24 h, MKG-48 h, WKG-24 h, and WKG-48 h) difficult. These variables include culture pH, TAA, and the concentrations of nutrients and fermentation products (8 variables); counts of the three microbial groups (3 variables); levels of vitamins (8 variables); mineral concentrations (10 variables); and the number of volatile compounds with an OAV ≥ 1.0 (1 variable).

**Figure 3:**
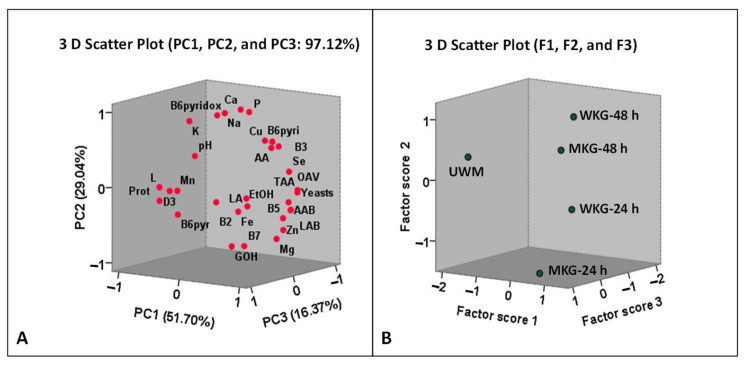
(**A**): Distribution of the independent variables (pH; counts of LAB, AAB, and yeasts; concentrations of lactose (L), proteins (Prot), lactic acid (LA), acetic acid (AA), ethanol (EtOH), and glycerol (GOH); minerals (K, Ca, P, Na, Mg, Zn, Fe, Mn, Cu, and Se); vitamins (B2, B3, B5, B6 (pyridoxal, B6pyr), B6 (pyridoxine, B6pyri), B6 (pyridoxamine, B6pyridox), B7, and D3); total antibacterial activity (TAA); and the number of volatile compounds with an OAV ≥ 1.0 [OAV]) as a function of PC1, PC2, and PC3. (**B**): Distribution of the UWM and the fermented beverages (MKG-24 h, MKG-48 h, WKG-24 h and WKG-48 h) as a function of Factor 1, Factor 2, and Factor 3.

With the application of PCA to these data, three principal components (PC1, PC2, and PC3) with eigenvalues greater than 1.0 were obtained, accounting for 51.70%, 29.04%, and 16.37% of the overall variance, respectively. Collectively, these components accounted for 97.12% of the overall variance (Figure 3A; Table S1).

PC1 was mainly associated with elevated levels of fermentation metabolites and microbial activity, including lactic acid, acetic acid, ethanol, and increased counts of LAB, AAB, and yeasts. It also showed positive correlations with vitamins B3, B5, and B6 (pyridoxine), Cu and Se concentrations, and the number of volatile compounds with an OAV ≥ 1.0 (Table S2). These variables reflect the biochemical transformations occurring during fermentation. PC2 was associated with mineral composition, particularly K, Ca, P, and Na, as well as vitamin B6 (pyridoxamine), whereas PC3 was primarily related to vitamin B2 and Mn levels (Table S2).

The distribution of the five beverages according to their corresponding factor scores (F1, F2, and F3) is presented in Figure 3B.

**Figure 4:**
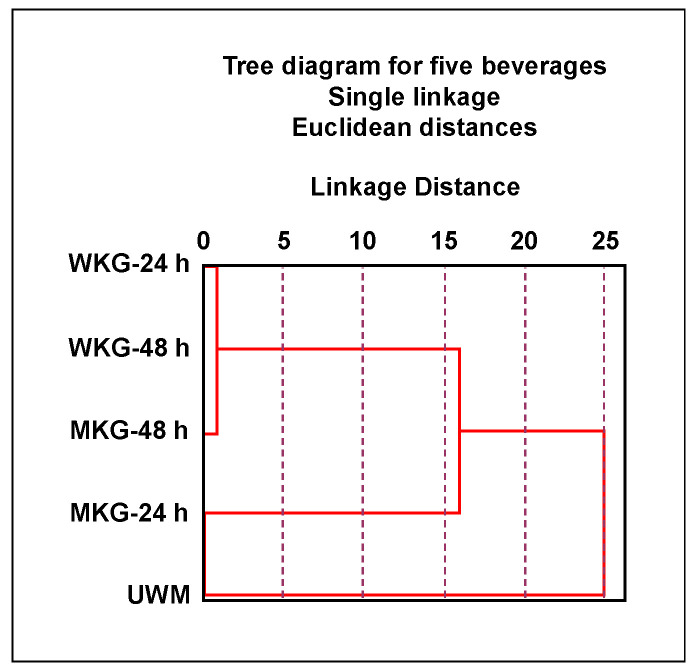
Cluster formed by the five milk beverages (UWM, MKG-24 h, MKG-48 h, WKG-24 h, and WKG-48 h) based on the three factor scores (F1, F2, and F3) obtained from the PCA.

To further explore similarities and differences among the samples, the three factor scores (F1, F2, and F3) obtained from PCA were used for cluster analysis (Figure 4). This analysis showed that WKG-24 h and WKG-48 h formed the first subcluster, with the lowest distance index (1.789), indicating a high degree of similarity between these beverages. Subsequently, MKG-48 h joined this group at a distance index of 1.800, forming a second subcluster. In contrast, MKG-24 h and UWM were sequentially incorporated at higher distance indices (2.213 and 2.478, respectively), indicating progressively greater differences from the previously formed clusters.

These results indicate that fermentation of whole milk with either MKGs or WKGs for 24 or 48 h produces beverages with distinct chemical, microbiological, and volatile profiles. The clustering pattern suggests that WKG-fermented beverages are more similar to each other than to those fermented with MKG. Moreover, the separation of MKG-24 h and UWM indicates that early fermentation stages still retain characteristics closer to the unfermented substrate.

These findings support the feasibility of producing fermented milk beverages using WKGs with biochemical and sensory characteristics that differ from those obtained with MKGs.

## 4. Conclusions

This study demonstrated that UHT whole milk can be successfully fermented using WKGs, although clear differences were observed in fermentation kinetics, microbial growth, chemical composition, and volatile profiles compared to MKGs. Accordingly, MKG fermentation showed more pronounced acidification and higher concentrations of lactic acid, ethanol, and glycerol, along with greater viable counts of the three microbial groups (LAB, AAB, and yeasts) compared with WKG fermentation.

Fermentation also modified the nutritional and aromatic characteristics of the beverages. Several B-group vitamins—particularly B2, B3, B5, and pyridoxine—increased during fermentation, especially in MKG samples, whereas vitamin D3 slightly decreased and the mineral composition remained relatively stable. The volatile profile was also affected, with MKGs promoting the formation of fatty acids, alcohols, and lactones associated with creamy and floral notes, whereas WKGs favored ester formation and fruity aromas.

Future studies should further characterize the microbial communities present in MKGs and WKGs and their corresponding beverages using advanced molecular techniques, investigate the adaptation of WKGs to milk through sequential subculturing, and evaluate the sensory properties and consumer acceptance of the fermented beverages to better assess their commercial potential.

## Figures and Tables

**Table 1:** Concentrations (µg/L) of vitamins (mean ± S.D. of two experiments with three analytical replicates each) identified in UWM and in samples fermented with MKG and WKG for 24 and 48 h.

No.	Vitamins	UWM	MKG-24 h	MKG-48 h	WKG-24 h	WKG-48 h
1	B2	239.65 ± 18.17 ^A^	262.28 ± 12.22 ^B,A^	277.55 ± 9.31 ^C,B^	206.28 ± 28.81 ^D,A^	215.40 ± 14.42 ^D^
2	B3	116.64 ± 35.30 ^A^	194.08 ± 11.47 ^B^	352.67 ± 4.89 ^C^	174.73 ± 15.17 ^D^	347.55 ± 3.75 ^C^
3	B5	1231.75 ± 293.80 ^A^	1735.65 ± 57.46 ^B^	1746.19 ± 75.29 ^B^	1589.25 ± 59.75 ^C^	1519.50 ± 2.12 ^A^
4	B6 (pyridoxal)	24.79 ± 5.01 ^A^	20.25 ± 2.16 ^B,A^	10.37± 1.27 ^C^	22.33 ± 0.82 ^A,B^	14.63 ± 2.70 ^D^
5	B6 (pyridoxine)	0.12 ± 0.00 ^A^	0.82 ± 0.15 ^B^	4.52 ± 0.23 ^C^	0.78 ± 0.24 ^B^	3.56 ± 0.34 ^D^
6	B6 (pyridoxamine)	2.41 ± 0.26 ^A^	1.54 ± 0.11 ^B^	2.63 ± 0.32 ^C,A^	1.66 ± 0.53 ^B^	2.49 ± 0.08 ^A,C^
7	B7	5.59 ± 1.45 ^A^	8.83 ± 1.02 ^B^	7.42 ± 0.88 ^C^	6.47 ± 0.48 ^A,C^	5.56 ± 0.33 ^A^
8	D3	162.70 ± 3.11 ^A^	132.19 ± 7.66 ^B^	111.38 ± 5.50 ^C^	145.00 ± 20.36 ^D,B^	127.50 ± 3.18 ^B,D^

Mean values within each row followed by different letters are significantly different (*p* < 0.05).

**Table 2:** Concentrations (mg/kg) of minerals (mean ± S.D. of two experiments with three analytical replicates each) identified in UWM and in WM fermented with MKG and WKG for 24 and 48 h.

No.	Mineral	UWM	MKG-24 h	MKG-48 h	WKG-24 h	WKG-48 h
1	K	1729.20 ± 93.70 ^A^	1544.38 ± 153.16 ^B,A^	1681.74 ± 29.56 ^C,A,B^	1573.66 ± 100.20 ^D,A,B,C^	1662.82 ± 91.75 ^A,B,C^
2	Ca	1186.31 ± 66.70 ^A^	1135.63 ± 100.55 ^B,A^	1206.78 ± 26.83 ^C,A,B^	1169.02 ± 70.58 ^D,A,B,C^	1225.21 ± 70.10 ^A,B,C^
3	P	941.51 ± 53.17 ^A^	908.50 ± 80.44 ^B,A^	965.42 ± 21.47 ^C,A,B^	935.21 ± 56.45 ^D,A,B,C^	980.17 ± 56.08 ^A,B,C^
4	Na	369.92 ± 26.63 ^A^	316.83 ± 53.44 ^A^	359.82 ± 9.36 ^A^	362.57 ± 16.81 ^A^	379.98 ± 26.92 ^A^
5	Mg	109.70 ± 17.14 ^A^	134.55 ± 31.52 ^A^	124.65 ± 13.52 ^A^	133.53 ± 17.56 ^A^	120.56 ± 5.97 ^A^
6	Zn	3.57 ± 0.56 ^A^	4.62 ± 0.77 ^A^	4.16 ± 0.41 ^A^	4.52 ± 0.91 ^A^	4.26 ± 0.17 ^A^
7	Fe	0.26 ± 0.00 ^A^	0.26 ± 0.00 ^A^	<0.20 ^B^	0.77 ± 0.00 ^C^	<0.20 ^B^
8	Mn	<0.20 ^A^	<0.20 ^A^	<0.20 ^A^	<0.20 ^A^	<0.20 ^A^
9	Cu	<0.20 ^A^	<0.20 ^A^	<0.20 ^A^	<0.20 ^A^	<0.20 ^A^
10	Se	0.26 ± 0.00 ^A^	0.26 ± 0.00 ^A^	0.26 ± 0.00 ^A^	0.26 ± 0.00 ^A^	0.26 ± 0.00 ^A^

Mean values within each row followed by different letters are significantly different (*p* < 0.05).

**Table 3:** Concentrations (mg/L) of VOCs (mean ± S.D. of two experiments with three analytical replicates each) identified in UWM and in samples fermented with MKG and WKG for 24 and 48 h.

No.	Compound	UWM	MKG-24 h	MKG-48 h	WKG-24 h	WKG-48 h
Organic Acids						
1	Pentanoic acid	N.d.	N.d.	0.93 ± 0.00 ^A^	N.d.	N.d.
2	Hexanoic acid	N.d.	0.79 ± 0.13 ^A^	2.53 ± 0.00 ^B^	N.d.	N.d.
3	Octanoic acid	2.55 ± 0.03 ^A^	3.72 ± 0.90 ^B,A^	5.69 ± 0.14 ^C^	2.03 ± 0.00 ^B^	5.23 ± 0.30 ^B,C^
4	Nonanoic acid	N.d.	N.d.	0.63 ± 0.14 ^A^	N.d.	0.43 ± 0.04 ^A^
5	n-Decanoic acid	2.83 ± 0.04 ^A^	4.99 ± 0.92 ^B^	11.47 ± 1.49 ^C^	5.89 ± 0.72 ^B^	12.74 ± 2.02 ^C^
6	Dodecanoic acid	0.82 ± 0.01 ^A^	0.95 ± 0.16 ^B,A^	1.94 ± 0.25 ^C^	1.32 ± 0.18 ^D,B,C^	2.73 ± 0.56 ^E^
7	Tetradecanoic acid	N.d.	N.d.	N.d.	N.d.	0.83 ± 0.16 ^A^
8	n-Hexadecanoic acid	N.d.	0.24 ± 0.00 ^A^	0.51 ± 0.19 ^B^	0.43 ± 0.16 ^B^	N.d.
	No. of Organic Acids	3	5	7	4	5
	Total	6.21 ± 1.18 ^A^	10.44 ± 1.90 ^B^	22.43 ± 3.81 ^C^	9.67 ± 1.98 ^D,B^	21.96 ± 4.32 ^C^
Alcohols						
9	2-Heptanol	N.d.	0.54 ± 0.04 ^A^	N.d.	0.38 ± 0.02 ^B^	0.24 ± 0.00 ^C^
10	2-(p-Tolyl)propan-2-ol	N.d.	0.20 ± 0.00 ^A^	0.24 ± 0.00 ^B^	N.d.	N.d.
11	2-Nonanol	N.d.	0.85 ± 0.11 ^A^	1.10 ± 0.08 ^B,A^	0.80 ± 0.00 ^C,A,B^	0.81 ± 0.11 ^A,B,C^
12	2-Dodecanol	N.d.	N.d.	N.d.	N.d.	0.40 ± 0.09 ^A^
13	Phenylethyl Alcohol	N.d.	0.30 ± 0.06 ^A^	1.70 ± 0.35 ^B^	N.d.	N.d.
14	Nerol	N.d.	N.d.	0.33 ± 0.00 ^A^	N.d.	0.21 ± 0.01 ^B^
15	E,E,Z-1,3,12-Nonadecatriene-5,14-diol	N.d.	N.d.	0.26 ± 0.03 ^A^	N.d.	N.d.
	No. of Alcohols	N.d.	4	5	2	4
	Total	N.d.	1.88 ± 0.31 ^A^	3.63 ± 0.62 ^B^	1.18 ± 0.30 ^C,A^	1.66 ± 0.28 ^A,C^
Ketones						
16	2-Heptanone	1.81 ± 0.01 ^A^	N.d.	N.d.	N.d.	N.d.
17	3-Octanone	0.36 ± 0.00 ^A^	0.32 ± 0.00 ^B^	N.d.	0.26 ± 0.00 ^C^	0.30 ± 0.03 ^A,B,C^
18	2-Nonanone	2.20 ± 0.04 ^A^	0.43 ± 0.10 ^B^	1.06 ± 0.00 ^C^	N.d.	N.d.
19	2-Undecanone	2.36 ± 0.03 ^A^	0.96 ± 0.04 ^B^	1.24 ± 0.06 ^C^	0.74 ± 0.04 ^D^	0.42 ± 0.02 ^E^
20	2-Tridecanone	0.96 ± 0.01 ^A^	0.46 ± 0.05 ^B^	0.75 ± 0.07 ^C^	0.43 ± 0.05 ^D,B^	0.44 ± 0.07 ^B,D^
	No. of Ketones	5	4	3	3	3
	Total	7.75 ± 0.80 ^A^	2.17 ± 0.33 ^B^	3.05 ± 0.55 ^B^	1.43 ± 0.29 ^D,B^	1.16 ± 0.21 ^D^
Esters						
21	Octanoic acid, ethyl ester	N.d.	0.93 ± 0.16 ^A^	0.24 ± 0.00 ^B^	4.65 ± 1.22 ^C^	4.11 ± 1.19 ^C^
22	Phthalic acid, di(2-propylpentyl) ester	N.d.	0.22 ± 0.00 ^A^	0.58 ± 0.17 ^B^	N.d.	N.d.
23	Decanoic acid, ethyl ester	N.d.	N.d.	0.29 ± 0.00 ^A^	0.53 ± 0.00 ^B^	0.38 ± 0.00 ^C^
24	Hexadecanoic acid, ethyl ester	N.d.	N.d.	N.d.	2.58 ± 0.00 ^A^	N.d.
	No. of Esters	N.d.	2	3	3	2
	Total	N.d.	1.15 ± 0.52 ^A^	1.12 ± 0.26 ^A^	7.76 ± 2.01 ^B^	4.50 ± 2.03 ^C^
Lactones						
25	δ-Decalactone	0.24 ± 0.00 ^A^	0.73 ± 0.12 ^B^	1.54 ± 0.09 ^C^	0.63 ± 0.06 ^D,B^	0.77 ± 0.10 ^B,D^
26	γ-Dodecalactone	0.48 ± 0.00 ^A^	0.37 ± 0.05 ^B,A^	0.64 ± 0.02 ^C^	0.35 ± 0.01 ^D,B^	0.45 ± 0.05 ^A,B,D^
27	δ-Dodecalactone	0.74 ± 0.01 ^A^	0.75 ± 0.13 ^B,A^	1.44 ± 0.02 ^C^	0.65 ± 0.01 ^D,B^	0.76 ± 0.13 ^A,B,D^
	No. of Lactones	3	3	3	3	3
	Total	1.46 ± 0.22 ^A^	1.86 ± 0.21 ^B,A^	3.62 ± 0.44 ^C^	1.64 ± 0.15 ^D,A,B^	1.97 ± 0.18 ^A,B,D^
Other Compounds						
28	Heptanediamide, N,N’-di-benzoyloxy-	N.d.	0.84 ± 0.13 ^A^	N.d.	N.d.	N.d.
29	D-Limonene	N.d.	0.42 ± 0.05 ^A^	1.06 ± 0.17 ^B^	0.32 ± 0.02 ^C,A^	0.30 ± 0.10 ^A,C^
30	Oxime-, methoxy-phenyl-	N.d.	N.d.	N.d.	0.22 ± 0.09 ^A^	0.21 ± 0.02 ^A^
31	Octanediamide, N,N’-di-benzoyloxy-	N.d.	N.d.	N.d.	2.12 ± 1.15 ^A^	3.15 ± 0.94 ^A^
	No. of Other Compounds	N.d.	2	1	3	3
	Total	N.d.	1.26 ± 0.25 ^A^	1.06 ± 0.17 ^B,A^	2.66 ± 1.08 ^C,A,B^	3.67 ± 1.55 ^A,B,C^

N.d.: not detected. Mean values within each row followed by different letters are significantly different (*p* < 0.05).

**Table 4:** OPT (mg/L), descriptors, and OAVs of VOCs identified in UWM and in milk fermented with MKG and WKG for 24 and 48 h.

				OAV				
No.	Compound	OPT	Descriptor	UWM	MKG-24 h	MKG-48 h	WKG-24 h	WKG-48 h
			Organic Acids					
1	Pentanoic acid	3 [81]	Green fruit, vegetables, sweat [82,83]	–	–	0.33 ± 0.00	–	–
2	Hexanoic acid	3 [81]	Goat cheese, fatty acids, vegetable oil, sweaty, sharp, acidic, green, gammy, dairy sour, dairy, stale, butter, sour, fruity, and pungent [84,85,86,87]	–	0.26 ± 0.14	0.84 ± 0.00	–	–
3	Octanoic acid	3 [81]	Sweat, creamy, cheese, rancid, fatty acids, vegetable oil [86,88,89]	0.85 ± 0.01	1.24 ± 0.30	1.90 ± 1.38	0.68 ± 0.00	1.74 ± 0.10
4	Nonanoic acid	8.8 [81]	Fatty, soapy, waxy, green, goat [90]	–	–	0.07 ± 0.01	–	0.05 ± 0.00
5	n-Decanoic acid	1 [91]	Waxy, fruity, cheese [11], fatty [91]	2.83 ± 0.04	4.99 ± 0.92	11.47 ± 1.49	5.89 ± 0.72	12.74 ± 2.02
6	Dodecanoic acid	2.2–16 [92]	Fatty/coconut/bay oil [93]	0.09 ± 0.00	0.10 ± 0.02	0.21 ± 0.03	0.14 ± 0.02	0.30 ± 0.06
7	Tetradecanoic acid	0.01 [81]	Cheese, greasy [88]	–	–	–	–	0.08 ± 0.02
8	n-Hexadecanoic acid	N.f.	Low heavy waxy, with a creamy, candle waxy nuance [94]	–	–	–	–	–
			Alcohols					
9	2-Heptanol	0.06523 [81]	Fresh [11], green [83,86]	–	8.22 ± 2.23	–	5.82 ± 0.38	3.74 ± 0.02
10	2-Nonanol	0.07 [53]	Green, fruity [95,96]	–	12.13 ± 159	15.77 ± 1.14	11.40 ± 0.03	11.51 ± 1.54
11	2-Dodecanol	0.041–0.082 [97]	Metallic [98]	–	–	–	–	6.48 ± 1.50
12	Phenylethyl alcohol	0.5642 [81]	Floral, pink, flowery, honey [86,99,100]	–	0.53 ± 0.11	3.02 ± 0.62	–	–
13	Nerol	0.5 [101]	Floral, green [101]	–	–	0.65 ± 0.00	–	0.43 ± 0.02
			Ketones					
14	2-Heptanone	0.005 [102]	Banana, fruity, floral and musty, fresh cream flavor [86,103,104]	362.38 ± 2.00	–	–	–	–
15	3-Octanone	0.028–0.050 [105]	Stale, moldy, old, slightly fruity, sweet, pear-like, candy-like, “cooked” [106]	9.25 ± 0.10	8.32 ± 0.11	–	6.56 ± 0.07	7.63 ± 0.68
16	2-Nonanone	0.005 [102]	Sweet, fruity, floral, musty [86,104]	440.63 ± 8.00	85.56 ± 20.27	213.00 ± 0.51	–	–
17	2-Undecanone	0.0055 [107]	Tallow, musty [107], fruity [108],	428.63 ± 5.45	174.32 ± 7.60	224.60 ± 10.93	133.98 ± 6.76	75.60 ± 2.94
18	2-Tridecanone	0.02–0.03 [109]	Tanning leather [109], heated milk-like [110]	38.24 ± 0.40	18.37 ± 2.16	29.92 ± 2.64	17.33 ± 1.90	17.79 ± 2.92
			Esters					
19	Octanoic acid, ethyl ester	0.0193 [81]	Fruity, apple, banana [111,112]	–	48.18 ± 44.44	12.69 ± 0.00	240.82 ± 63.09	213.02 ± 113.59
20	Decanoic acid, ethyl ester	0.023 [81]	Apple, floral, fruity, and musty [111,113,114]	–	–	12.64 ± 0.04	23.23 ± 0.00	16.73 ± 0.00
21	Hexadecanoic acid, ethyl ester	2 [115]	Fruity, creamy, waxy [116]	–	–	–	1.29 ± 0.00	–
			Lactones					
22	δ-Decalactone	0.0025 [117]	Creamy [118], coconut [117]	96.96 ± 1.86	292.83 ± 46.65	616.11 ± 36.38	253.13 ± 22.82	306.13 ± 39.03
23	γ-Dodecalactone	0.007 [101]	Creamy [118], coconut, fruity-sweet [101], dairy, floral, honey, body milk [119]	69.13 ± 0.43	53.48 ± 7.34	91.69 ± 0.02	50.29 ± 1.43	63.76 ± 7.05
24	δ-Dodecalactone	0.0046 [120]	Coconut, cheesy, creamy, sweet, fruity [118], fatty [101]	160.19 ± 2.17	164.02 ± 28.98	312.67 ± 4.32	142.20 ± 3.03	165.28 ± 27.29
Other Compounds								
25	D-Limonene	0.01 [121]	Citrus, mint [121], pine/herbal/peppery [93], fruity, lemon [110],	–	42.44 ± 4.52	106.38 ± 16.76	32.41 ± 1.62	30.10 ± 9.79
Compounds with OVA ≥ 1.0		–	–	9	13	13	13	14

Mean OAVs are presented; N.f.: not found; “–” indicates not detected. Odor descriptors and OPT references are provided for each compound.

## Data Availability

The data supporting the findings of this study are available within the article and its Supplementary Materials. Further inquiries can be directed to the corresponding author.

## Supplementary Materials

The following supporting information can be downloaded at: https://www.mdpi.com/article/10.3390/foods15101616/s1, Table S1: Total variance explained by the principal component analysis (PCA) of the five milk beverages (UWM, MKG-24 h, MKG-48 h, WKG-24 h, and WKG-48 h), based on pH; microbiological counts (lactic acid bacteria, acetic acid bacteria, and yeasts); concentrations of lactose and fermentation metabolites (lactic acid, acetic acid, ethanol, and glycerol); minerals (K, Ca, P, Na, Mg, Zn, Fe, Mn, Cu, and Se); vitamins (B2, B3, B5, B6 (pyridoxal), B6 (pyridoxine), B6 (pyridoxamine), B7, and D3); total antibacterial activity; and the number of volatile compounds with OAV ≥ 1.0. Table S2: Component matrix obtained by principal component analysis (PCA) of the five milk beverages (UWM, MKG-24 h, MKG-48 h, WKG-24 h, and WKG-48 h), based on their chemical and microbiological composition, vitamin and mineral contents, and the number of volatile compounds (VOCs) with OAV ≥ 1.0.

## References

[1] Leite A.M.O., Miguel M.A., Peixoto R.S., Rosado A.S., Silva J.T., Paschoalin V.M. (2013). Microbiological, technological and therapeutic properties of kefir: A natural probiotic beverage. Braz. J. Microbiol..

[2] Bourrie B.C.T., Willing B.P., Cotter P.D. (2016). The microbiota and health promoting characteristics of the fermented beverage kefir. Front. Microbiol..

[3] Farag M.A., Jomaa S.A., El-Wahed A.A., El-Seedi H.R. (2020). The many faces of kefir fermented dairy products: Quality characteristics, flavour chemistry, nutritional value, health benefits, and safety. Nutrients.

[4] Fijan S., Bržan P.P., Pogačar M.Š., Klanjšek P. (2026). Kefir consumption and health effects based on human clinical trials: An overview of literature. Healthcare.

[5] Rafie N., Golpour Hamedani S., Ghiasvand R., Miraghajani M. (2015). Kefir and cancer: A systematic review of literatures. Arch. Iran. Med..

[6] Abajo-Justel M., Balvis-Outeiriño E., Pérez-Guerra N. (2026). Production of kefir and kefir-like beverages: Fundamental aspects, advances, and future challenges. Processes.

[7] Cais-Sokolińska D., Wójtowski J., Pikul J., Danków R., Majcher M., Teichert J., Bagnicka E. (2015). Formation of volatile compounds in kefir made of goat and sheep milk with high polyunsaturated fatty acid content. J. Dairy Sci..

[8] Kavas G. (2015). Kefirs manufactured from camel (*Camelus dromedarius*) milk and cow milk: Comparison of some chemical and microbial properties. Ital. J. Food Sci..

[9] Rutkowska J., Antoniewska-Krzeska A., Zbikowska A., Cazón P., Vázquez M. (2022). Volatile composition and sensory profile of lactose-free kefir, and its acceptability by elderly consumers. Molecules.

[10] Li X., Zhao Z., Shi S., Li D., Sang Y., Wang P., Zhao L., Wang F., Fang B., Chen S. (2024). Flavor properties of post-heated fermented milk revealed by a comprehensive analysis based on volatile and non-volatile metabolites and sensory evaluation. Curr. Res. Food Sci..

[11] Ströher J.A., Oliveira W.d.C., Freitas A.S., Salazar M.M., Flôres S.H., Malheiros P.d.S. (2025). Microbial dynamics and volatile compound profiles in artisanal kefir during storage. Fermentation.

[12] Otles S., Çağındı O. (2003). Kefir: A probiotic dairy–composition, nutritional and therapeutic aspects. Pak. J. Nutr..

[13] Zongo O., Cruvellier N., Leray F., Bideaux C., Lesage J., Zongo C., Traoré Y., Savadogo A., Guillouet S. (2020). Physicochemical composition and fermentation kinetics of a novel palm sap-based kefir beverage from the fermentation of *Borassus aethiopum* Mart. fresh sap with kefir grains and ferments. Sci. Afr..

[14] Bensmira M., Jiang B. (2015). Total phenolic compounds and antioxidant activity of a novel peanut-based kefir. Food Sci. Biotechnol..

[15] Sabokbar N., Khodaiyan F., Moosavi-Nasab M. (2014). Optimization of processing conditions to improve antioxidant activities of apple juice and whey-based novel beverage fermented by kefir grains. J. Food Sci. Technol..

[16] Sabokbar N., Moosavi-Nasab M., Khodaiyan F. (2015). Preparation and characterization of an apple juice and whey-based novel beverage fermented using kefir grains. Food Sci. Biotechnol..

[17] Bazán D.L., del Río P.G., Domínguez J.M., Cortés-Diéguez S., Mejuto J.C., Pérez-Guerra N. (2022). The chemical, microbiological and volatile composition of kefir-like beverages produced from red table grape juice in repeated 24-h fed-batch subcultures. Foods.

[18] Bazán D.L., Del-Río P.G., Pérez-Guerra N. (2025). Microbiological and chemical profiles of kiwi kefir-like beverages produced using different agitation speeds and kefir grain weights. Foods.

[19] Afonso M.J., Ramalhosa E., del Río P.G., Martins F., Baptista P., Pereira E.L., Guerra N.P. (2025). Production of nondairy fermented products with chestnut puree as substrate and milk kefir grains or two lactic acid bacteria. J. Food Sci..

[20] Fiorda F.A., Pereira G.V.M., Thomaz-Soccol V., Medeiros A.P., Rakshit S.K., Soccol C.R. (2016). Development of kefir-based probiotic beverages with DNA protection and antioxidant activities using soybean hydrolyzed extract, colostrum and honey. LWT-Food Sci. Technol..

[21] Koh W.Y., Utra U., Rosma A., Effarizah M., Rosli W.I.W., Park Y.H. (2018). Development of a novel fermented pumpkin-based beverage inoculated with water kefir grains: A response surface methodology approach. Food Sci. Biotechnol..

[22] Guzel-Seydim Z.B., Gökırmaklı C., Greene A.K. (2021). A comparison of milk kefir and water kefir: Physical, chemical, microbiological and functional properties. Trends Food Sci. Technol..

[23] Bazán D.L., Del-Río P.G., Cortés Diéguez S., Domínguez J.M., Pérez Guerra N. (2024). Main composition and visual appearance of milk kefir beverages obtained from four consecutive 24- and 48-h batch subcultures. Processes.

[24] Corona O., Randazzo W., Miceli A., Guarcello R., Francesca N., Erten H., Moschetti G., Settanni L. (2016). Characterization of kefir-like beverages produced from vegetable juices. LWT-Food Sci. Technol..

[25] Lowry O.H., Rosebrough N.J., Farr A.L., Randall R.J. (1951). Protein measurement with the Folin phenol reagent. J. Biol. Chem..

[26] Warakaulle S., Mohamed H., Ranasinghe M., Shah I., Yanyang X., Chen G., Ayyash M.M., Vincent D., Kamal-Eldin A. (2024). Advancement of milk protein analysis: From determination of total proteins to their identification and quantification by proteomic approaches. J. Food Compos. Anal..

[27] Turker N., Kizilkaya S., Cevik R. (2013). The mineral composition of kefir produced from goat and cow milk. J. Food Agric. Environ..

[28] Ferreira N., Henriques B., Viana T., Carvalho L., Tavares D., Pinto J., Jacinto J., Colonia J., Pereira E. (2023). Validation of a methodology to quantify macro, micro, and potentially toxic elements in food matrices. Food Chem..

[29] Gliszczyńska-Świgło A., Rybicka I. (2021). Fast and sensitive method for phosphorus determination in dairy products. J. Consum. Prot. Food Saf..

[30] Gentili A., Caretti F., D’Ascenzo G., Marchese S., Perret D., Di Corcia D., Mainero Rocca L. (2008). Simultaneous determination of water-soluble vitamins in selected food matrices by liquid chromatography/electrospray ionization tandem mass spectrometry. Rapid Commun. Mass. Spectrom..

[31] Stevens J., Dowell D. (2012). Determination of vitamins D2 and D3 in infant formula and adult nutritionals by ultra-pressure liquid chromatography with tandem mass spectrometry detection (UPLC-MS/MS): First Action 2011.12. J. AOAC Int..

[32] Cabo M.L., Murado M.A., González M.P., Pastoriza L. (1999). A method for bacteriocin quantification. J. Appl. Microbiol..

[33] Murado M.A., González M.P., Vázquez J.A. (2002). Dose–response relationships: An overview, a generative model and its application to the verification of descriptive models. Enzym. Microb. Technol..

[34] Guerra N.P. (2025). Enhancing logistic modeling for diauxic growth and biphasic antibacterial activity synthesis by lactic acid bacteria in realkalized fed-batch fermentations. Mathematics.

[35] Magalhães K.T., de Melo Pereira G.V., Campos C.R., Dragone G., Schwan R.F. (2011). Brazilian kefir: Structure, microbial communities and chemical composition. Braz. J. Microbiol..

[36] Gamba R.R., Yamamoto S., Abdel-Hamid M., Sasaki T., Michihata T., Koyanagi T., Enomoto T. (2020). Chemical, microbiological, and functional characterization of kefir produced from cow’s milk and soy milk. Int. J. Microbiol..

[37] Alves E., Ntungwe E.N., Gregório J., Rodrigues L.M., Pereira-Leite C., Caleja C., Pereira E., Barros L., Aguilar-Vilas M.V., Rosado C. (2021). Characterization of kefir produced in household conditions: Physicochemical and nutritional profile, and storage stability. Foods.

[38] Leite A.M., Leite D.C., Del Aquila E.M., Alvares T.S., Peixoto R.S., Miguel M.A., Silva J.T., Paschoalin V.M. (2013). Microbiological and chemical characteristics of Brazilian kefir during fermentation and storage processes. J. Dairy Sci..

[39] Kök-Taş T., Seydim A.C., Özer B., Güzel-Seydim Z.B. (2013). Effects of different fermentation parameters on quality characteristics of kefir. J. Dairy Sci..

[40] Tada S., Katakura Y., Ninomiya K., Shioya S. (2007). Fed-batch coculture of Lactobacillus kefiranofaciens with *Saccharomyces cerevisiae* for effective production of kefiran. J. Biosci. Bioeng..

[41] Kirtadze E., Nutsubidze N. (2009). Metabolic potential of alcoholic fermentation yeasts. Bull. Georgian Natl. Acad. Sci..

[42] Cheirsilp B., Radchabut S. (2011). Use of whey lactose from dairy industry for economical kefiran production by *Lactobacillus kefiranofaciens* in mixed cultures with yeast. New Biotechnol..

[43] Meng Y., Wang X., Li Y., Chen J., Chen X. (2024). Microbial interactions and dynamic changes of volatile flavor compounds during the fermentation of traditional kombucha. Food Chem..

[44] Oude Elferink S.J.W.H., Krooneman J., Gottschal J.C., Spoelstra S.F., Faber F., Driehuis F. (2001). Anaerobic conversion of lactic acid to acetic acid and 1,2-propanediol by *Lactobacillus buchneri*. Appl. Environ. Microbiol..

[45] Liu S.Q. (2003). Practical implications of lactate and pyruvate metabolism by lactic acid bacteria in food and beverage fermentations. Int. J. Food Microbiol..

[46] Pintado J., Raimbault M., Guyot J. (2005). Influence of polysaccharides on oxygen-dependent lactate utilization by an amylolytic *Lactobacillus plantarum* strain. Int. J. Food Microbiol..

[47] Freire A.L., Ramos C.L., de Almeida E.G., Duarte W.F., Schwan R.F. (2014). Study of the physicochemical parameters and spontaneous fermentation during the traditional production of Yakupa, an indigenous beverage produced by Brazilian Amerindians. World J. Microbiol. Biotechnol..

[48] Puerari C., Magalhães-Guedes K.T., Schwan R.F. (2015). Physicochemical and microbiological characterization of chicha, a rice-based fermented beverage produced by Umutina Brazilian Amerindians. Food Microbiol..

[49] Axelsson L., Salminen S., Wright A.V., Ouwehand A. (2004). Lactic acid bacteria: Classification and physiology. Lactic Acid Bacteria: Microbiological and Functional Aspects.

[50] Puerari C., Magalhães K.T., Schwan R.F. (2012). New cocoa pulp-based kefir beverages: Microbiological, chemical composition, and sensory analysis. Food Res. Int..

[51] Arai H., Sakurai K., Ishii M., Matsushita K., Toyama H., Tonouchi N., Okamoto-Kainuma A. (2016). Metabolic features of *Acetobacter aceti*. Acetic Acid Bacteria.

[52] De Vuyst L., Comasio A., Van Kerrebroeck S. (2023). Sourdough production: Fermentation strategies, microbial ecology, and use of non-flour ingredients. Crit. Rev. Food Sci. Nutr..

[53] Gomes R.J., Borges M.F., Rosa M.F., Castro-Gómez R.J.H., Spinosa W.A. (2018). Acetic acid bacteria in the food industry: Systematics, characteristics and applications. Food Technol. Biotechnol..

[54] Gul O., Mortas M., Atalar I., Dervisoglu M., Kahyaoglu T. (2015). Manufacture and characterization of kefir made from cow and buffalo milk using kefir grain and starter culture. J. Dairy Sci..

[55] Yilmaz L., Özcan Yilsay T., Akpinar Bayizit A. (2006). The sensory characteristics of berry-flavoured kefir. Czech. J. Food Sci..

[56] Magalhães K.T., de Melo Pereira G.V., Dias D.R., Schwan R.F. (2010). Microbial communities and chemical changes during fermentation of sugary Brazilian kefir. World J. Microbiol. Biotechnol..

[57] Dikmetas D.N., Acar E.G., Ceylan F.D., İlkadım F., Özer H., Karbancioglu-Guler F. (2025). Functional fermented fruit juice production and characterization by using water kefir grains. J. Food Sci. Technol..

[58] Gao X., Li B. (2016). Chemical and microbiological characteristics of kefir grains and their fermented dairy products: A review. Cogent Food Agric..

[59] Moradi Z., Kalanpour N. (2019). Kefiran, a branched polysaccharide: Preparation, properties and applications—A review. Carbohydr. Polym..

[60] Irigoyen A., Arana I., Castiella M., Torre P., Ibáñez F.C. (2005). Microbiological, physicochemical, and sensory characteristics of kefir during storage. Food Chem..

[61] Arroum S., Sboui A., Fguiri I., Dbara M., Ayeb N., Hammadi M., Khorchani T. (2025). Influence of kefir grain concentration on the nutritional, microbiological, and sensory properties of camel milk kefir and characterization of some technological properties. Fermentation.

[62] Satir G., Guzel-Seydim Z.B. (2016). How kefir fermentation can affect product composition?. Small Rumin. Res..

[63] Tawfek M.A., Baker E.A., El-Sayed H.A. (2021). Study properties of fermented camels’ and goats’ milk beverages fortified with date palm (*Phoenix dactylifera* L.). Food Nutr. Sci..

[64] Randazzo W., Corona O., Guarcello R., Francesca N., Germanà M.A., Erten H., Moschetti G., Settanni L. (2016). Development of new non-dairy beverages from Mediterranean fruit juices fermented with water kefir microorganisms. Food Microbiol..

[65] FAO/WHO (2006). Probiotic in foods. Health and nutritional properties and guidelines for evaluation. FAO Food and Nutrition.

[66] Raeisi S.N., Ouoba L.I.I., Farahmand N., Sutherland J., Ghoddusi H.B. (2013). Variation, viability and validity of bifidobacteria in fermented milk products. Food Control.

[67] Mirdula D., Sharma M. (2015). Development of non-dairy probiotic drink utilizing sprouted cereals, legume and soymilk. LWT-Food Sci. Technol..

[68] Sayes C., Leyton Y., Riquelme C., Savić S. (2018). Probiotic bacteria as a healthy alternative for fish aquaculture. Antibiotic Use in Animals.

[69] Gallina D.A., Menezes Barbosa P.P., Celeste Ormenese R.C.S., Garcia A.O. (2019). Development and characterization of probiotic fermented smoothie beverage. Rev. Cienc. Agron..

[70] LeBlanc J.G., Taranto M.P., Molina V., Sesma F., Mozzi F., Raya R., Vignolo G. (2010). B-group vitamins production by probiotic lactic acid bacteria. Biotechnology of Lactic Acid Bacteria: Novel Applications.

[71] LeBlanc J.G., Laiño J.E., Juarez del Valle M., Vannini V., van Sinderen D., Taranto M.P., Font de Valdez G., Savoy de Giori G., Sesma F. (2011). B-group vitamin production by lactic acid bacteria: Current knowledge and potential applications. J. Appl. Microbiol..

[72] Linares D.M., Fitzgerald G., Hill C., Stanton C., Ross P., Tamime A.Y., Thomas L.V. (2017). Production of vitamins, exopolysaccharides and bacteriocins by probiotic bacteria. Probiotic Dairy Products.

[73] Santos Júnior V., Nizoli E., Galvan D., Gomes R.J., Biz G., Ressutte J.B., Rocha T.S., Spinosa W.A. (2022). Micronutrient Requirements and Effects on Cellular Growth of Acetic Acid Bacteria Involved in Vinegar Production. Food Sci. Technol..

[74] Kessi-Pérez E.I., González A., Palacios J.L., Martínez C. (2022). Yeast as a Biological Platform for Vitamin D Production: A Promising Alternative to Help Reduce Vitamin D Deficiency in Humans. Yeast.

[75] Evers M.S., Ramousse L., Morge C., Sparrow C., Gobert A., Roullier-Gall C., Alexandre H. (2023). To Be or Not to Be Required: Yeast Vitaminic Requirements in Winemaking. Food Microbiol..

[76] Liutkevicius A., Sarkinas A. (2004). Studies on the growth conditions and composition of kefir grains as a food and forage biomass. Vet. Zootech..

[77] Arslan S. (2015). A review: Chemical, microbiological and nutritional characteristics of kefir. CyTA—J. Food.

[78] Prado M.R., Blandón L.M., Vandenberghe L.P.S., Rodrigues C., Castro G.R., Thomaz-Soccol V., Soccol C.R. (2015). Milk kefir: Composition, microbial cultures, biological activities, and related products. Front. Microbiol..

[79] Muñoz-Bas C., Muñoz-Tebar N., Viuda-Martos M., Lucas-González R., Pérez-Álvarez J.Á., Fernández-López J. (2025). Quality properties of innovative goat milk kefir enriched with date paste (*Phoenix dactylifera* L.) and whey derived from goat cheese production. Foods.

[80] Rosa D.D., Dias M.M.S., Grześkowiak Ł.M., Reis S.A., Conceição L.L., Peluzio M.C.G. (2017). Milk kefir: Nutritional, microbiological and health benefits. Nutr. Res. Rev..

[81] van Gemert L.J. (2011). Compilations of Odour Threshold Values in Air, Water and Other Media.

[82] Czerny M., Schieberle P. (2002). Important aroma compounds in freshly ground whole meal and white wheat flour: Identification and quantitative changes during sourdough fermentation. J. Agric. Food Chem..

[83] Thomsen M., Martin C., Mercier F., Tournayre P., Berdagué J.L., Thomas-Danguin T., Guichard E. (2012). Investigating semi-hard cheese aroma: Relationship between sensory profiles and gas chromatography–olfactometry data. Int. Dairy J..

[84] Escudero A., Gogorza B., Melús M.A., Ortín N., Cacho J.A., Ferreira V. (2004). Characterization of the aroma of a wine from Maccabeo: Key role played by compounds with low odor activity values. J. Agric. Food Chem..

[85] Dertli E., Çon A.H. (2017). Microbial diversity of traditional kefir grains and their role in kefir aroma. LWT—Food Sci. Technol..

[86] Li B., Gao X., Li N., Mei J. (2018). Fermentation process of mulberry juice-whey based Tibetan kefir beverage production. Czech. J. Food Sci..

[87] Pereira R., Resende D., Alencar A.C., de Abreu L.R., Ferreira W. (2019). Survival of Kluyveromyces lactis and Torulaspora delbrueckii to simulated gastrointestinal conditions and their use as single and mixed inoculum for cheese production. Food Res. Int..

[88] Ning M., He K., Zhang D., Liu S., Zhang Y., Min J., Wu R., Wu J., Zhang S. (2025). HS-SPME-GC-MS combined with multivariate statistical analysis reveals off-flavor composition and biomarker generation mechanism of whole milk powder. J. Food Compos. Anal..

[89] Oliveira D.R., Lopes A.C.A., Pereira R.A., Cardoso P.G., Duarte W.F. (2019). Selection of potentially probiotic Kluyveromyces lactis for the fermentation of cheese whey-based beverage. Ann. Microbiol..

[90] Walsh A.M., Crispie F., Kilcawley K., O’Sullivan O., O’Sullivan M.G., Claesson M.J., Cotter P.D. (2016). Microbial succession and flavor production in the fermented dairy beverage kefir. mSystems.

[91] Gil M., Cabellos J.M., Arroyo T., Prodanov M. (2006). Characterization of the volatile fraction of young wines from the denomination of origin “Vinos de Madrid” (Spain). Anal. Chim. Acta.

[92] Molimard P., Spinnler H.E. (1996). Review: Compounds involved in the flavor of surface mold-ripened cheeses: Origins and properties. J. Dairy Sci..

[93] Durán-Guerrero E., Castro R., García-Moreno M.V., Rodríguez-Dodero M.C., Schwarz M., Guillén-Sánchez D. (2021). Aroma of sherry products: A review. Foods.

[94] Sfakianakis P., Tzia C. (2017). Flavour profiling by gas chromatography–mass spectrometry and sensory analysis of yoghurt derived from ultrasonicated and homogenised milk. Int. Dairy J..

[95] Welke J.E., Zanus M., Lazzarotto M., Alcaraz C. (2014). Quantitative analysis of headspace volatile compounds using comprehensive two-dimensional gas chromatography and their contribution to the aroma of Chardonnay wine. Food Res. Int..

[96] de March C.A., Ryu S.E., Sicard G., Moon C., Golebiowski J. (2015). Structure–odour relationships reviewed in the postgenomic era. Flavour Fragr. J..

[97] Schnabel K.O., Belitz H.D., von Ranson C. (1988). Investigations on the structure–activity relationships of odorous substances. Part 1. Detection thresholds and odour qualities of aliphatic and alicyclic compounds containing oxygen functions. Z. Lebensm. Unters. Forsch..

[98] Chastrette M., Cretin D., El Aïdi C. (1996). Structure–odor relationships: Using neural networks in the estimation of camphoraceous or fruity odors and olfactory thresholds of aliphatic alcohols. J. Chem. Inf. Comput. Sci..

[99] Hayaloglu A.A., Brechany E.Y., Deegan K.C., McSweeney P.L.H. (2008). Characterization of the chemistry, biochemistry and volatile profile of Kuflu cheese, a mould-ripened variety. LWT—Food Sci. Technol..

[100] Zheng X., Li K., Shi X., Ni Y., Li B., Zhuge B. (2018). Potential characterization of yeasts isolated from Kazak artisanal cheese to produce flavoring compounds. MicrobiologyOpen.

[101] Tong W., Zhai H., Qi M., Hua Y., Shi T., Shang H., Shi Y., Duan C., Lan Y. (2024). Characterization of chemical and sensory properties of Cabernet Sauvignon and Marselan wines made by flash détente technique. Food Res. Int..

[102] Dan T., Chen H., Li T., Tian J., Ren W., Zhang H., Sun T. (2019). Influence of *Lactobacillus plantarum* P-8 on fermented milk flavor and storage stability. Front. Microbiol..

[103] Tian H., Shi Y., Zhang Y., Yu H., Mu H., Chen C. (2019). Screening of aroma-producing lactic acid bacteria and their application in improving the aromatic profile of yogurt. J. Food Biochem..

[104] Dimitrellou D., Kandylis P., Lević S., Petrović T., Ivanović S., Nedović V., Kourkoutas Y. (2019). Encapsulation of Lactobacillus casei ATCC 393 in alginate capsules for probiotic fermented milk production. LWT—Food Sci. Technol..

[105] Callejón R.M., Ubeda C., Ríos-Reina R., Morales M.L., Troncoso A.M. (2016). Recent developments in the analysis of musty odour compounds in water and wine: A review. J. Chromatogr. A.

[106] Siegmund B., Pöllinger-Zierler B. (2006). Odor thresholds of microbially induced off-flavor compounds in apple juice. J. Agric. Food Chem..

[107] Giri A., Osako K., Ohshima T. (2010). Identification and characterisation of headspace volatiles of fish miso, a Japanese fish meat-based fermented paste. Food Chem..

[108] Cheng Z., O’Sullivan M.G., Miao S., Kerry J.P., Kilcawley K.N. (2022). Sensorial, cultural and volatile properties of milk, dairy powders, yoghurt and butter: A review. Int. J. Dairy Technol..

[109] Gibka J. (2004). Odor characteristics of aliphatic metameric C-13 ketones, alcohols and their derivatives. Perfum. Flavorist.

[110] Moio L., Piombino P., Addeo F. (2000). Odour-impact compounds of Gorgonzola cheese. J. Dairy Res..

[111] Liu S.Q., Holland R., Crow V.L. (2004). Esters and their biosynthesis in fermented dairy products: A review. Int. Dairy J..

[112] Cheng H. (2010). Volatile flavor compounds in yogurt: A review. Crit. Rev. Food Sci. Nutr..

[113] Sánchez-Palomo E., Trujillo M., García-Ruiz A., González-Viñas M.A. (2017). Aroma profile of Malbec red wines from La Mancha region: Chemical and sensory characterization. Food Res. Int..

[114] Nikolaou A., Tsakiris A., Kanellaki M., Bezirtzoglou E., Akrida-Demertzi K., Kourkoutas Y. (2019). Wine production using free and immobilized kefir culture on natural supports. Food Chem..

[115] Salgado J.M., González C., Rodríguez R., Simal J., Domínguez J.M., Cortés S. (2012). Study of the volatile compounds produced by *Debaryomyces hansenii* NRRL Y-7426 during the fermentation of detoxified concentrated distilled grape marc hemicellulosic hydrolysates. World J. Microbiol. Biotechnol..

[116] Liang J., Yoo M.J.Y., Seale B., Grazioli G. (2021). Nutritional and volatile characterisation of milk inoculated with thermo-tolerant *Lactobacillus bulgaricus* through adaptive laboratory evolution. Foods.

[117] Ong P.K.C., Acree T.E. (1999). Similarities in the aroma chemistry of Gewürztraminer variety wines and lychee (*Litchi chinensis* Sonn.) fruit. J. Agric. Food Chem..

[118] Chen C., Liu Z., Yu H., Xu Z., Tian H. (2022). Flavoromic determination of lactones in cheddar cheese by GC–MS–olfactometry, aroma extract dilution analysis, aroma recombination and omission analysis. Food Chem..

[119] Ríos-Reina R., Segura-Borrego M.P., Morales M.L., Callejón R.M. (2020). Characterization of the aroma profile and key odorants of Spanish PDO wine vinegars. Food Chem..

[120] Karagul-Yuceer Y., Vlahovich K.N., Drake M., Cadwallader K.R. (2003). Characteristic aroma components of rennet casein. J. Agric. Food Chem..

[121] Risner D., Tomasino E., Hughes P., Meunier-Goddik L. (2019). Volatile aroma composition of distillates produced from fermented sweet and acid whey. J. Dairy Sci..

[122] Tian H., Shen Y., Yu H., He Y., Chen C. (2017). Effects of 4 probiotic strains in coculture with traditional starters on the flavor profile of yogurt. J. Food Sci..

[123] Knight M.J., Bull I.D., Curnow P. (2014). The yeast enzyme Eht1 is an octanoyl-CoA: Ethanol acyltransferase that also functions as a thioesterase. Yeast.

[124] Alam M.K., Prete R., Rannou C., Lethuaut L., Faieta M., Perla C., Prost C., Pittia P., Corsetti A. (2025). Microbial impact on the volatolomic profile of fermented milks: A case study with the application of industrial starter cultures. Appl. Food Res..

[125] Gandy A.L., Schilling M.W., Coggins P.C., White C.H., Yoon Y., Kamadia V.V. (2008). The effect of pasteurization temperature on consumer acceptability, sensory characteristics, volatile compound composition, and shelf-life of fluid milk. J. Dairy Sci..

