# Supplementary material for: Production and Characterization of Kefir Beverages by Fermentation of Whole Milk with Milk or Water Kefir Grains
Source: Foods. 2026 May 7;15(10):1616. doi: 10.3390/foods15101616 (PMC13205963; doi:10.3390/foods15101616)
Supplement: Supplementary file 1 [file foods-15-01616-s001.zip › foods-4271635-supplementary.pdf]

Table S1. Total variance explained by the principal component analysis (PCA) of the five milk beverages (UWM, MKG-24 h, MKG-48 h, WKG-24 h, and WKG-48 h), based on pH; microbiological counts (lactic acid bacteria, acetic acid bacteria, and yeasts); concentrations of lactose and fermentation metabolites (lactic acid, acetic acid, ethanol, and glycerol); minerals (K, Ca, P, Na, Mg, Zn, Fe, Mn, Cu, and Se); vitamins (B2, B3, B5, B6 (pyridoxal), B6 (pyridoxine), B6 (pyridoxamine), B7, and D3); total antibacterial activity; and the number of volatile compounds with OAV ≥ 1.0.

	Initial Eigenvalues			Extraction Sums of Squared Loadings		
Component	Total	Variance (%)	Cumulative (%)	Total	Variance (%)	Cumulative (%)
1	15.511	51.704	15.7042	15.511	51.704	51.704
2	8.713	29.044	80.748	8.713	29.044	80.748
3	4.911	16.369	97.117	4.911	16.369	97.117

Extraction method: Principal component analysis.

Table S2. Component matrix obtained by principal component analysis (PCA) of the five milk beverages (UWM, MKG-24 h, MKG-48 h, WKG-24 h, and WKG-48 h), based on their chemical and microbiological composition, vitamin and mineral contents, and the number of volatile compounds (VOCs) with OAV ≥ 1.0.

	Component		
**Variable**	**PC1**	**PC2**	**PC3**
Lactose	-0.961	-0.111	0.206
Lactic acid	0.747	-0.033	0.587
Acetic acid	0.788	0.567	0.055
Ethanol	0.760	-0.137	0.581
Glycerol	0.431	-0.717	0.482
Proteins	-0.924	-0.280	0.254
pH	-0.851	0.217	-0.478
Total antibacterial activity	0.986	-0.153	-0.069
LAB count	0.939	-0.275	-0.188
AAB count	0.937	-0.281	-0.187
Yeasts count	0.932	-0.068	-0.355
B2	0.452	-0.070	0.880
B3	0.803	0.567	-0.097
B5	0.927	-0.365	-0.023
B6 (pyridoxal)	-0.858	-0.506	-0.084
B6 (pyridoxine)	0.768	0.641	0.003
B6 (pyridoxamine)	0.046	0.958	0.278
B7	0.580	-0.710	0.399
D3	-0.978	-0.200	-0.061
K	-0.353	0.854	0.364
Ca	0.117	0.977	-0.177
P	0.222	0.947	-0.230
Na	-0.301	0.854	-0.400
Mg	0.591	-0.722	-0.350
Zn	0.635	-0.613	-0.447
Fe	-0.257	-0.489	-0.654
Mn	-0.115	0.041	0.990
Cu	0.733	0.669	0.128
Se	0.897	0.224	-0.209
No. of VOC with with OAV ≥ 1.0	0.829	-0.071	-0.507

Extraction method: Principal component analysis.
